# SLOW: A novel spectral editing method for whole‐brain MRSI at ultra high magnetic field

**DOI:** 10.1002/mrm.29220

**Published:** 2022-03-28

**Authors:** Guodong Weng, Piotr Radojewski, Sulaiman Sheriff, Claus Kiefer, Philippe Schucht, Roland Wiest, Andrew A. Maudsley, Johannes Slotboom

**Affiliations:** ^1^ Institute for Diagnostic and Interventional Neuroradiology, Support Center for Advanced Neuroimaging (SCAN) University of Bern Bern Switzerland; ^2^ Department of Radiology University of Miami School of Medicine Miami Florida USA; ^3^ Department of Neurosurgery Inselspital Bern and University Hospital Bern Switzerland

**Keywords:** ^1^H MRSI, 7T, adiabatic pulse, chemical selective, J‐difference editing, whole‐brain

## Abstract

**Purpose:**

At ultra‐high field (UHF), B_1_^+^‐inhomogeneities and high specific absorption rate (SAR) of adiabatic slice‐selective RF‐pulses make spatial resolved spectral‐editing extremely challenging with the conventional MEGA‐approach. The purpose of the study was to develop a whole‐brain resolved spectral‐editing MRSI at UHF (UHF, *B*_0_ ≥ 7T) within clinical acceptable measurement‐time and minimal chemical‐shift‐displacement‐artifacts (CSDA) allowing for simultaneous GABA/Glx‐, 2HG‐, and PE‐editing on a clinical approved 7T‐scanner.

**Methods:**

Slice‐selective adiabatic refocusing RF‐pulses (2π‐SSAP) dominate the SAR to the patient in (semi)LASER based MEGA‐editing sequences, causing large CSDA and long measurement times to fulfill SAR requirements, even using SAR‐minimized GOIA‐pulses. Therefore, a novel type of spectral‐editing, called SLOW‐editing, using two different pairs of phase‐compensated chemical‐shift selective adiabatic refocusing‐pulses (2π‐CSAP) with different refocusing bandwidths were investigated to overcome these problems.

**Results:**

Compared to conventional echo‐planar spectroscopic imaging (EPSI) and MEGA‐editing, SLOW‐editing shows robust refocusing and editing performance despite to *B*_1_^*+*^‐inhomogeneity, and robustness to *B*_0_‐inhomogeneities (0.2 ppm ≥ Δ*B*_0_ ≥ −0.2 ppm). The narrow bandwidth (∼0.6–0.8 kHz) CSAP reduces the SAR by 92%, RF peak power by 84%, in‐excitation slab CSDA by 77%, and has no in‐plane CSDA. Furthermore, the CSAP implicitly dephases water, lipid and all the other signals outside of range (≥ 4.6 ppm and ≤1.4 ppm), resulting in additional water and lipid suppression (factors ≥ 1000s) at zero SAR‐cost, and no spectral aliasing artifacts.

**Conclusion:**

A new spectral‐editing has been developed that is especially suitable for UHF, and was successfully applied for 2HG, GABA+, PE, and Glx‐editing within 10 min clinical acceptable measurement time.

## 1 INTRODUCTION

Due to better SNR, localized MR spectroscopy benefits from ultra‐high field (UHF *B*_0_ ≥ 7T). In practice, however, at UHF many hurdles related to the underlying physics must be overcome.^1^ The first to be mentioned is the quadratic dependence of the specific absorption rate (SAR) of the electromagnetic RF‐field *B*_1_^+^ on the magnetic field strength^2^ (∝*B*_0_^2^), which results in a disproportionate tissue heating at higher fields. Additionally, the wavelength of the electromagnetic (EM) wave at UHF is most often shorter than the size of the anatomic structures to be examined: for instance, approximately 11 cm at 7T. This results in interference patterns (inhomogeneities) in RF‐field *B*_1_^+^, and inhomogeneous spectroscopic images. The effect of these *B*_1_^+^‐inhomogeneities can partially be overcome using adiabatic RF pulses^2^ or with parallel‐transmit (pTx) techniques.^3^ An additional consideration is the relatively low maximum reachable RF peak power of the high‐power RF‐amplifiers in commercial MR scanners, resulting in low reachable maximum *B*_1_^+^‐amplitudes, which drastically limits the available RF bandwidth. Although, as mentioned above, spatial‐selective adiabatic refocusing pulses can be used to overcome *B*_1_^+^‐inhomogeneities problem, they impose a high SAR burden for the patient. Therefore, the number of these pulses used should be kept as low as possible in any UHF MR pulse sequence. One method of reducing the SAR is to increase the TR of the MR pulse sequence, but this can make the measurement time too long for the patient study. Due to the above‐mentioned factors, the real practical available in vivo RF‐bandwidth (BW) (Δω_RF,max_) is very low, which leads to large chemical‐shift displacement‐artifact (CSDA), also known as chemical‐shift displacement error (CSDE) that scale with ∝1/Δω_RF,max_.^4^ An additional complicating factor in UHF MR spectroscopy applications is that a larger Δω_RF_ is needed compared to low‐field, in order to cover the full chemical‐shift range of all metabolites to be excited and refocused (denoted by Δω_spins_). More specific, this is because Δω_spins_∝*B*_0_. The combination of the maximum tolerable SAR in vivo, and the wider spectral‐bandwidth Δω_spins_ to be covered, makes spatially resolved MR spectroscopy at UHF extremely challenging.

Spectral editing^5^, ^6^ refers to a collection of NMR techniques that enable the selective detection of metabolites that are obscured by more‐intense overlapping resonances or strong nearby resonance(s). These techniques include multiple‐quantum editing techniques (e.g., for lactate editing^7^) and J‐difference editing techniques applied to GABA editing,^8^ 2‐hydroxyglutarate (2HG)^9^, ^10^ editing, and as most recently shown, phosphoethanolamine (PE)^11^, ^12^ editing. In in vivo applications, spectral‐editing techniques are combinations of volume‐localization schemes, such as semiLASER (Localization by Adiabatic SElective Refocusing),^4^, ^13^ with additional so‐called narrow band MEGA‐editing refocusing pulses, which is realized by adding MEGA‐editing pulses^8^ to the sequence. Most MEGA‐editing implementations use pure amplitude‐modulated Gaussian‐shaped refocusing‐pulses,^14^, ^15^, ^16^ for which the editing‐performance at UHF is degraded due to the above mentioned *B*_1_^+^‐inhomogeneities. A possible solution to improve the editing‐performance can be found in applying parallel‐transmit techniques (pTx).^17^ However, the use of pTx is not FDA approved yet, and therefore cannot be applied in clinical routine yet. Furthermore, *B*_0_ inhomogeneities for whole‐brain MRSI are even worse at UHF, which, together with *B*_1_^+^ inhomogeneities, negatively affect the spectral‐editing efficiency causing signal loss.^18^ To improve the editing performance, editing using adiabatic editing pulses is proposed here as an alternative to pTx. Recently, an editing technique using an adiabatic MEGA‐editing scheme was developed within a 1D‐semiLASER sequence combined with CONCEPT readout^18^ enabling single‐slice GABA edited MRSI (acquisition matrix, 32 × 32; TA = 24 min). Another developed flexible MEGA editing with 3D‐semiLASER technique using conventional spectral‐readout demonstrated highly efficient editing‐performance at 3T.^19^

In this report, we present a fully adiabatic spectral editing method, called SLOW. SLOW consists of a chemical‐shift selective adiabatic 2π‐pulse (CSAP) pair that covers nearly the complete 1/2 J time (with J being the scalar‐coupling constant of the spin‐system to be edited), without the need of additional slice‐selection refocusing pulses as is the case in MEGA‐semiLASER or MEGA‐PRESS. This approach not only solves the above‐described issues with limited *B*_1_^+^‐peak power and *B*_0_/*B*_1_^+^ inhomogeneities at UHF.

Furthermore, 2π‐CSAP does not introduce in‐plane CSDA and has *implicit* water and lipid suppression at zero additional SAR cost, which is an additional novel aspect. SLOW‐editing is as straightforward as MEGA‐editing and can be combined with any pulse sequence/readout scheme. To obtain lowest possible SAR, SLOW‐editing was built into an echo‐planar spectroscopic imaging (EPSI) sequence^20^ and enables straightforward whole‐brain editing of all, currently known, important brain‐metabolites (i.e., 2HG, GABA, PE, Glx). Due to the low SAR of this approach, short TRs can be used, and within a total measurement‐time of less than 10 min, whole‐brain 2HG/GABA+/PE/Glx SLOW‐edited MRSI datasets can be obtained on a Siemens 7T‐Terra system in *clinical* mode using the Nova 1Tx 32Rx head coil. The notion 1Tx refers to one transmit‐channel operating the RF‐coil in circular polarized, or CP, mode.

## 2 METHODS

### 2.1 2π‐CSAP‐EPSI

**FIGURE 1:**
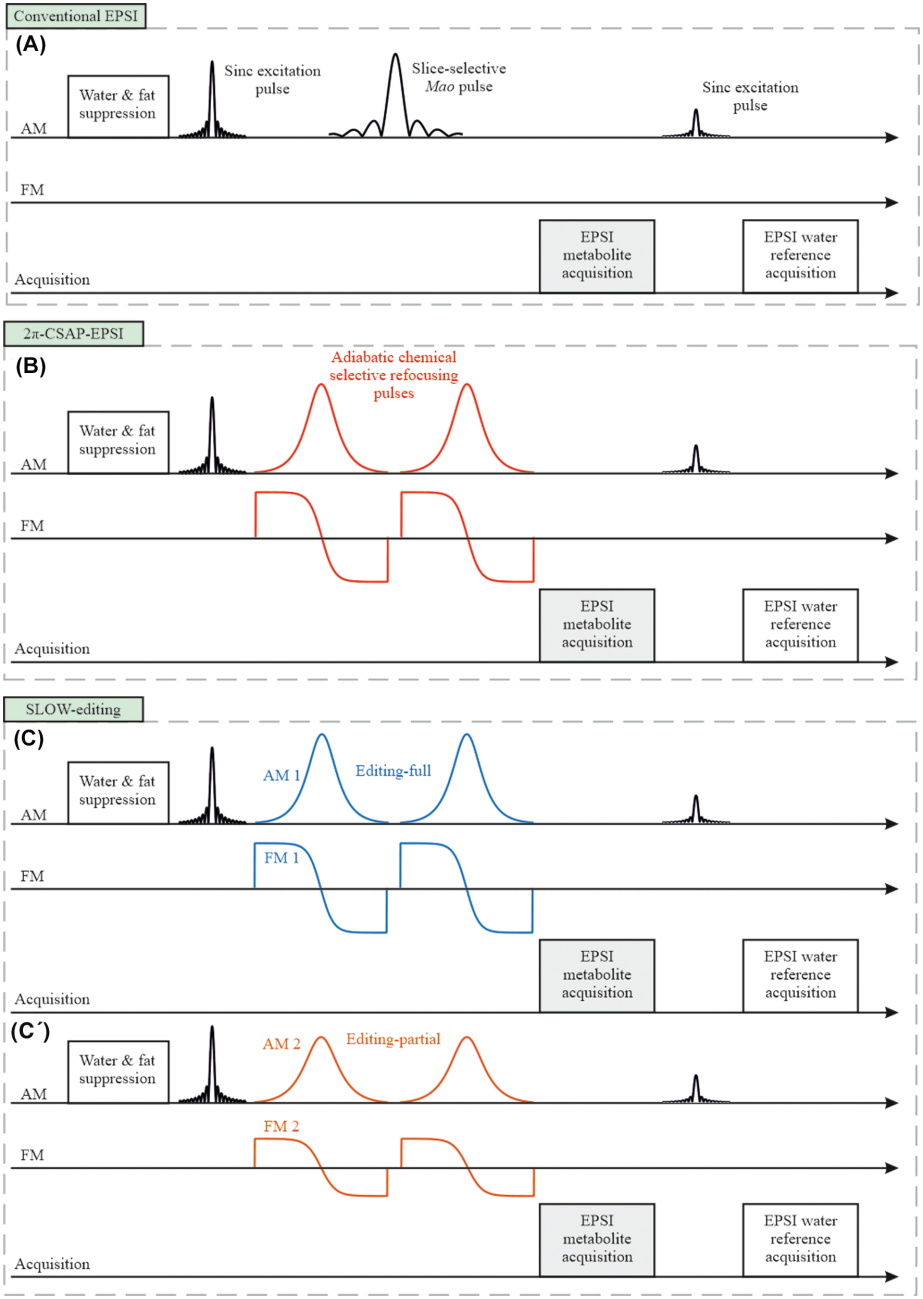
The sequence schemes. A, Traditional EPSI sequence makes use of a slice selective amplitude‐modulated refocusing pulse. B, The proposed new 2π‐CSAP‐EPSI sequence is using a CSAP pair. SLOW spectral‐editing sequence scheme integrated into EPSI is based on the scheme displayed in (A). C, Chemical selectively refocusing the full offset frequency range of the J‐coupled spin system to be edited. C′, Chemical selectively refocusing the partial range of interested spins

Figure 1A,B shows the adapted EPSI‐pulse sequence, in which the original slice‐selective refocusing *Mao* pulse^21^ was replaced by a phase compensated chemical‐shift selective adiabatic complex secant hyperbolic RF‐pulse pair $B_{1}\left(t\right)=\Omega_{0}\cdot \mathrm{sech}{\left(\beta t\right)}^{1+\mu i}$, further denoted as 2π‐CSAP.^22^ Specifically, the bandwidth of 2π‐CSAP pulses was typically set to 0.81 kHz (i.e., ∼2.7 ppm at *B*_0_ = 7T) which covers all major metabolites, and the carrier frequency was set at 3.0 ppm. For chemical‐selective refocusing, as proposed here, we need narrow bandwidths *but* steep transition bands. In this sense, the choice of complex secant hyperbolic RF‐pulse is motivated by the fact that this RF‐pulse shape has smaller transition band compared to WURST‐16 RF‐pulse type^23^, ^24^ for comparable pulse duration (Supporting Information Figures S16 and S17, which are available online).

### 2.2 SLOW‐editing

Based on the 2π‐CSAP‐EPSI‐sequence, we propose a novel spectral‐editing approach, referred to as SLOW (SLOtboom‐Weng). SLOW is realized by selectively refocusing *two* different offset frequency ranges mimicking editing “off” and “on” of MEGA‐type editing (Figure 1C,C′). In SLOW‐editing, we refer “editing‐off” as “editing‐full” and “editing‐on” as “editing‐partial.” That is, we acquire two datasets with editing‐full and editing‐partial, respectively. Subtraction of the two datasets yields the so‐called edited J‐difference spectrum. A more detailed description is given below.

### 2.3 Pulse design

RF‐pulse design of the 2π‐CSAP pulse‐sequences, as well as the quantum mechanical metabolite spectrum simulations, are performed using in‐house MATLAB code, by solving the relaxation‐free Liouville‐von Neumann equation.^4^

#### 2.3.1 The excitation pulse

The sinc‐Gaussian excitation pulse as following where BW f = 5500, b = 400 and t is [−3, 3] ms was defined by:

$$B_{1}\left(t\right)=\frac{\pi }{2}\cdot \frac{\sin\left(\pi \mathrm{tf}\right)}{\sin\left(\pi t\right)}\cdot \exp\left(-b^{2}t^{2}\right)$$

#### 2.3.2 The adiabatic pulses

The pulse functions are as follows^22^:

$$B_{1}\left(t\right)=\Omega_{0}\cdot \mathrm{sech}{\left(\beta t\right)}^{1+\mu i}$$

The parameter‐settings for different pulse‐schemes are shown on the Supporting Information Table S1.

### 2.4 Sequence design

As is shown in Supporting Information Figure S1, the sequence was developed based on EPSI‐sequence^20^ using the Siemens IDEA‐VE12U programming environment and it consists of the following parts:

1. Inversion‐recovery lipid‐suppression was realized by an asymmetric chemical‐shift selective adiabatic pulse, 120 Hz BW, 100 ms duration, and the carrier‐frequency is set to 1.57 ppm to suppress lipid‐contamination near NAA. The inversion‐time (TI) is 234 ms, which was measured in our 7T scanner with a lipid‐phantom. This asymmetric chemical‐shift selective adiabatic pulse is used to suppress *only* lipids near NAA (1.58–1.78 ppm), similar approach was reported in reference.^25^ Although this lipid suppression pulse is applied to the 2HG_1.83_ resonances, this pulse does not influence the editing results of 2HG_4.01_. Further details are presented in the Supporting Information Materials in section “IR lipid suppression pulse” (Supporting Information Figures S2 and S3).
2. Water‐suppression was realized by five amplitude‐modulated Gaussian‐pulses that were numerically optimized for optimal performance despite *B*_1_^+^‐inhomogeneities (like the WET^26^ technique, but without the need of T1‐insensitive consideration). The flip angles are 78‐24‐82‐35‐75 degrees. The pulse‐duration is 24.32 ms, and the time‐interval between each pulse is 14.4 ms. The spoiler gradient has an amplitude of 12.17 mT/m and 13.8 ms duration.
3. Excitation, refocusing, and editing: A slice‐selective sinc‐Gaussian pulse with 6 ms duration, 5.5 kHz BW, and a 65‐degree Ernst flip angle is used to maximize the signal for excitation. The CSAP‐pair is used for *both* refocusing and editing, which is described in detail in the main text of the paper. The spoiler gradient pairs are placed directly adjacent to the two adiabatic‐pulses. The gradient durations are 1.9‐1.9‐3.0‐3.0 ms and having following amplitudes of 8.84‐8.84‐16.8‐16.8 mT/m and 8.84‐8.84‐1.87‐1.87 mT/m in X‐ and Z‐axes, respectively.
4. EPSI readout.^20^ The readout is composed by 2048 gradient lobes which generates 1024 even and odd echoes. The ramp time, duration, and amplitude of each gradient lobe are set to be 190 μs, 390 μs, and 19.92 mT/m, respectively. This is followed by a spoiler gradient with 20 ms duration and 5 mT/m amplitude. For all in vivo measurement, the carrier frequency was set to be 3 ppm instead of 4.7 ppm of water.
5. Water reference excitation. The same slice‐selective sinc‐Gaussian pulse as in part 3 is used, but with a flip angle of 10 degrees, and followed by a gradient‐echo readout in part 6.
6. Water reference EPSI‐readout.^20^ The same readout‐scheme as in part 4.

### 2.5 Sequence parameters

#### 2.5.1 2π‐CSAP‐EPSI

TE = 82 ms, TR = 1500 or 1551 ms, one average. Vector‐size = 1024, *B*_0_ Shim mode = Advanced, preparation‐scans = 5, phase encoding = Elliptical. Excitation and adiabatic‐pulses carrier‐frequency and acquisition carrier‐frequency = 3 ppm. The acquisition sweep‐bandwidth = 1.28 kHz. The typical matrix for in vivo measurement matrix is 65 × 25 × 15 (4.3 × 7.2 × 7.3 mm), and measurement time = 8:31 min.

#### 2.5.2 SLOW‐editing scheme 1 (2HG)

This scheme is only used for detection of the 2HG at 1.88 ppm in vitro and should illustrate the SLOW‐editing working principle (Figure 4A).

TE = 120 ms, TR = 1500 ms. Editing‐full and partial pulses carrier frequencies are 3 and 2 ppm, respectively. The typical matrix for in vitro measurement is 65 × 23 × 9 (4.3 × 7.8 × 7.8 mm), and measurement‐time = 9:04 min. Other sequence‐parameters are as stated above.

#### 2.5.3 SLOW‐editing scheme 2 (GABA/Glx/2HG)

This scheme can detect the GABA, Glx, and 2HG signal at 3.00, 3.75, and 4.01 ppm, respectively, with TE = 68 ms, TR = 1500 ms (Figure 4A). Editing‐full and ‐partial pulses carrier frequencies are 2.90 and 3.45 ppm, respectively. The typical matrix for in vivo measurement is 65 × 23 × 9 (4.3 × 7.8 × 7.8 mm), and measurement‐time = 9:04 min. The other parameters are the same as in SLOW‐editing scheme 1.

#### 2.5.4 SLOW‐editing scheme 3 (PE)

This scheme can optimally detect the PE and Glx signal at 3.26 and 2.11 ppm, respectively, with TE = 90 ms, TR = 1500 ms (Figure 4A). Editing‐full and ‐partial pulses carrier frequencies are 3.00 and 2.60 ppm, respectively, and other parameters as in editing scheme 1.

### 2.6 MR scanner and head coil

Clinically approved MAGNETOM Terra 7T MR‐scanner (Siemens, Germany), Nova Medical Head Coil 1TX / 32RX, and 8TX/32RX (USA).

### 2.7 Phantoms

(1) Braino phantom (General Electric, USA), (2) spherical 2HG‐phantom prepared in‐house (∼7.8 mmol/L of 2HG and 18 mmol/L of glycine), and (3) spherical GABA‐phantom prepared in‐house (∼10 mmol/L of GABA, creatine, and glycine) as shown in Figure S4.

### 2.8 Patients and volunteers

Two patients and six volunteers were recruited and measured. This study was approved by the local ethical committee of Bern, Switzerland.

### 2.9 Reconstruction and pre‐post‐processing

The reconstruction and pre‐post‐processing were processed via Metabolic Imaging Data Analysis System (MIDAS),^27^ spectrIm‐QMRS and MATLAB R2019b. Further details were described in Supporting Information.

## 3 RESULTS

### 3.1 Chemical‐shift selective adiabatic refocusing

#### 3.1.1 Water‐suppression

**FIGURE 2:**
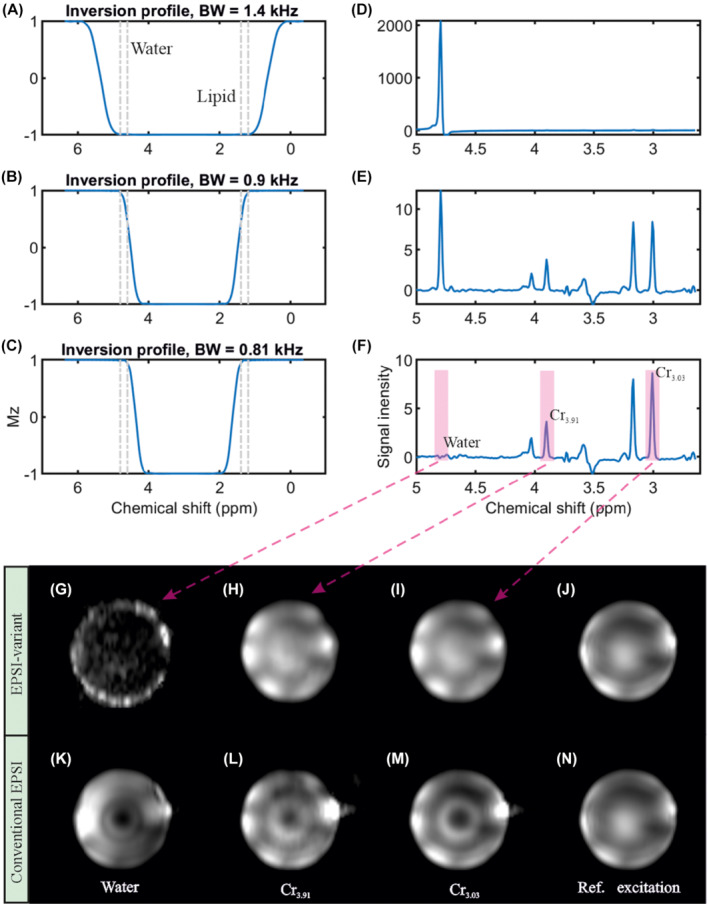
RF‐pulse simulation and phantom measurement. A–C, The simulated RF‐pulse profiles of the 2π‐CSAP (used as inversion pulse in the simulation for simplicity). D–F, The corresponding brain metabolite phantom measurements. G, The noise‐like water map, which emphasizes a superior homogeneous overall water suppression compared to (I). The two‐creatine (H‐I) and water reference (J) maps agree with each other, while (L‐N) show clearly different patterns. This proves that the proposed 2π‐CSAP guarantee a uniform refocusing in both the selected chemical shift dimension as well as in the complete excited spatial volume. The measurements were performed with TE = 82 ms, TR = 1500 ms, matrix = 65 × 20 × 8 (4.3 × 11 × 13.8 mm), and measurement time = 3 min

Three different adiabatic 2π‐CSAP with variable bandwidth Δω_RF_ were applied to investigate their performance on a spherical phantom at *B*_0_ = 7T (Figure 2A–C) with respect to their water‐suppression behavior. In these cases, TE = 82 ms, TR = 1500 ms, Δω_RF_ = 0 .81–1.4 kHz, matrix‐size = 65 × 20 × 8 (4.3 × 11 × 13.8 mm), and measurement‐time of 3 min. As shown in Figure 2, the 2π‐CSAP pulse‐parameters were chosen such that, in situation (a) the water‐resonance was completely in the pulses' passband; in situation (b) the water resonance was in the transition‐band; and in situation (c) the water resonance was in the stop band, respectively. In all three cases, the metabolite offset‐range was refocused. Water‐suppression can be accomplished by refocusing only the range of metabolites (1.8–4.2 ppm), as shown in Figure 2C. Figure 2F shows that nearly perfect water‐suppression is achieved with *additional* water signal‐suppression factors of >1000 s compared to Figure 2D. In addition, because of the symmetry of the refocused offset‐range about 3.0 ppm the same suppression‐performance is expected in vivo for the lipid‐region (0.9–1.3 ppm). Similar water‐suppression factors are observed in vivo, as will be shown below. Again, it should be noted that the implicit water and lipid suppression is obtained at *zero* additional SAR‐cost, which is a very important property for in vivo application at UHF. Near to ideal water suppression is also obtained in vivo, while lipid suppression near the skull is not perfect as is shown in Supporting Information Figures S10 and S11.

#### 3.1.2 Sensitivity to *B*_1_^+^ ‐inhomogeneities

To study the sensitivity to *B*_1_^+^‐homogeneities on performance of the refocusing pulses, between the 2π‐CSAP and the *Mao* refocusing pulse,^21^ two measurements were performed using the sequences shown in Figure 1A,B. No water‐ and fat‐suppression preparation pulses were applied. The noise‐like water map proves the superior implicit water suppression of 2π‐CSAP usage (Figure 2G). The consistency between creatine CH_2_ (3.91 ppm), CH_3_ (3.03 ppm), and the water reference peak integration maps (Figure 2H–J) proves the robustness of the 2π‐CSAP with respect to the *B*_1_^+^‐inhomogeneity. In contrast, Figure 2K–N show the same maps obtained with the *Mao* refocusing pulse; these maps show a strongly inhomogeneous signal distribution, especially toward the center of the spherical phantom, indicating the same inhomogeneous *B*_1_^+^ distribution when applying the *Mao* pulse.

**FIGURE 3:**
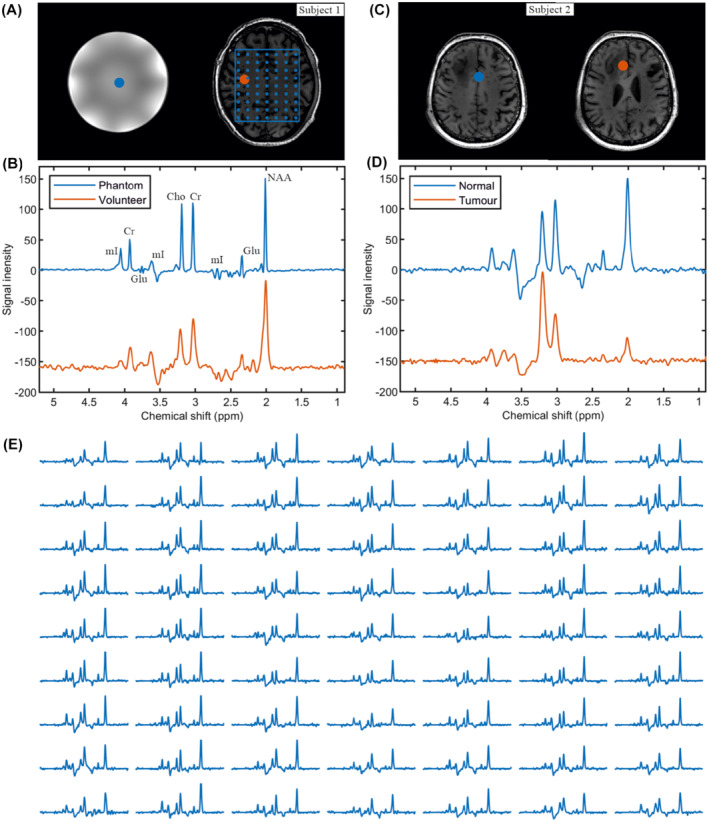
In vitro and in vivo measurement. A, T1‐weighted MRI of a Braino phantom and healthy volunteer. B, 2π‐CSAP‐EPSI of the Braino phantom (see the Methods section) and healthy volunteer where the selected volumes are indicated with blue and orange dots. The 2π‐CSAP‐EPSI is performed with TE = 82 ms, TR = 1551 ms, BW = 0.81 kHz, matrix = 65 × 25 × 15 (4.3 × 7.2 × 7.3 mm), and measurement time = 8:31 min. C,D, T1‐weighted and 2π‐CSAP‐EPSIs of a patient with currently unconfirmed tumor‐type, with the selected volumes marked with blue and orange dots in the normal and tumor areas, respectively. The 2π‐CSAP‐EPSI was performed with TE = 82 ms, TR = 1551 ms, BW = 0.81 kHz, matrix = 65 × 28 × 14 (4.3 × 7.9 × 7.9 mm), and measurement time = 7:41 min. E, The array spectrum plots of selected area (indicated by blue line and dots in A). The different size of the blue and orange dots does not represent the different size of the displaced voxels, which is the same for all of them (4.3 × 4.3 × 7.9 mm)

In vivo studies of a healthy volunteer and a brain‐tumor patient (Figure 3B,D) show a high degree of agreement with the in vitro measurement using a Braino‐phantom (Figure 3B). The sequence was performed with TE = 82 ms, TR = 1551 ms, BW = 0.81 kHz, matrix = 65 × 25 × 15 (4.3 × 7.2 × 7.3 mm), and measurement time = 8:31 min. The spectrum within the tumor region shows a clearly different, typical brain tumor pattern than the normal tissue of the same subject (Figure 3D). That is, the tumor NAA (2.008 ppm) and creatine (3.03 ppm) signals are smaller than in normal tissue.

#### 3.1.3 Chemical shift displacement artifacts

The original EPSI‐implementation used a slice‐selective *Mao* refocusing pulse which had a Δω_RF_ = 1.25 kHz and was limited by scanners' maximum obtainable RF amplitude. In the slice selection direction, the CSDA per ppm of the *Mao* refocusing is 297/1250 = 23.7% per ppm (1 ppm at 7T equals 297 Hz). In our 2π‐CSAP case, the CSDA is only determined by the excitation pulse (Δω_RF_ = 5500 Hz) and is only 297/5500 = 5.4% per ppm. Therefore, the overall CSDA error is reduced by approximately 1.0–5.4/23.7 = 77%. Since there are no spatial‐selective gradients that apply in both the X‐ and Y‐dimensions, no in‐plane CSDA is generated. The very low CSDA by using 2π‐CSAP pulses makes the interpretation of the spectra much more straightforward, since there is no in plane CSDA; only in the two most peripheral slices the CSDA has a small effect.

#### 3.1.4 SAR and peak power

The SAR of similarly shaped RF pulses is proportional to BW, and the RF peak power is proportional to $\sqrt{\mathrm{BW}/\tau }$, with *τ* being the pulse duration. Therefore, assuming the same adiabatic condition, the comparison between 2π‐CSAP (duration = 31 ms, BW = 0.81 kHz) and the hyperbolic secant adiabatic refocusing pluses in normal MEGA‐semiLASER/semiLASER (duration = 5 ms, BW = 5.3 kHz)^16^ is as follows. The SAR of 2π‐CSAP is approximately 7.6% (0.81 kHz/[2 × 5.3 kHz]) of the *two pairs* refocusing pulses of semiLASER, while the RF peak power is about 15.7% ($\sqrt{\left(0.81\;\mathrm{kHz}/31\;\mathrm{ms}\right)/\left(5.3\;\mathrm{kHz}/5\;\mathrm{ms}\right)}$).

#### 3.1.5 Spectral quality and post‐processing

For the results obtained with 2π‐CSAP in this paper, *only* k‐space re‐gridding, 4D‐Fourier transformation, apodization filtering, and baseline correction were performed *without* the need of additional water removal. In contrast, additional water removal was necessary in all cases when using the *Mao* refocusing pulses. Although the spectral quality is already very good with this minimal post‐processing, there are still various possibilities to improve the results by post‐processing, such as corrections for Δ*B*_0_, eddy‐current correction (ECC) and corrections for transmit (*B*_1_^+^) inhomogeneity and receive (*B*_1_^−^) sensitivity.

### 3.2 Spectral editing

#### 3.2.1 SLOW‐editing schemes

**FIGURE 4:**
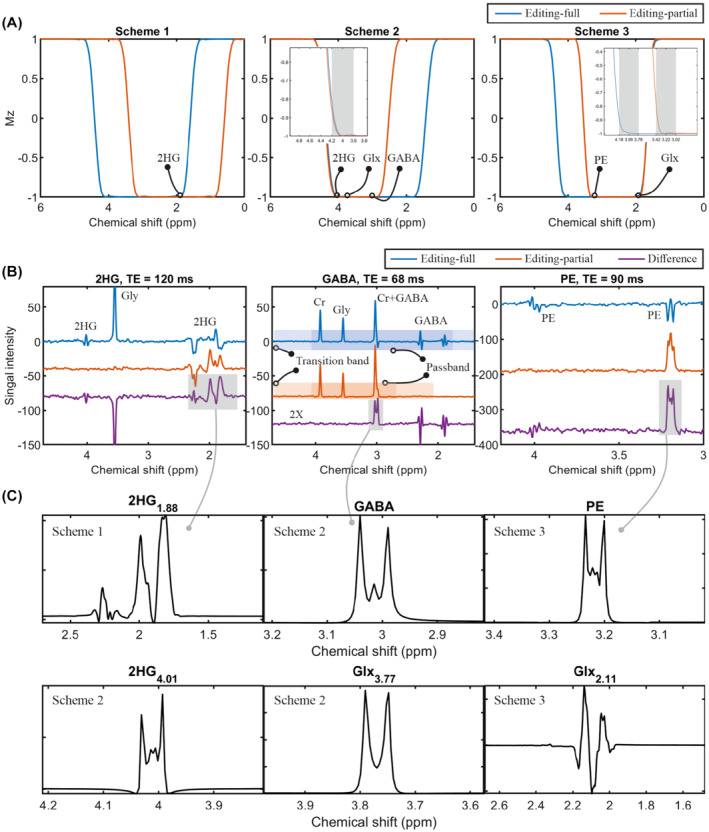
SLOW‐editing schemes with phantom measurements and simulations. A, Simulation of the adiabatic pulse (used as inversion pulse in the simulation for simplicity) in three editing schemes: Scheme 1 for 2HG_4.01_ editing; scheme 2 for GABA editing (inserted figure with finer scale), and scheme 3 for PE editing (inserted figure with finer scale). B, In vitro measurements to detect 2HG, GABA, and PE using editing scheme 1, 2, and 3, respectively. The pass band and transition‐band are indicated by blue/orange and light blue/orange for editing‐full and editing‐partial pulses, respectively. TE = 120 ms (2HG), 68 ms (GABA), and 90 ms (PE), TR = 1500 ms, spatial resolution = 4.3 × 4.3–11 × 11–18.3 mm, and total measurement time less than 10 min. C, The corresponding metabolites simulation with three editing schemes (2HG_1.88_ for scheme 1; 2HG_4.01_, GABA, and Glx_3.77_ [glutamate/glutamine = 2:1] for scheme 2; PE and Glx_2.11_ [glutamate/glutamine = 2:1] for scheme 3). The TE‐s are 120, 68, and 90 ms, respectively

SLOW‐editing further uses the superior properties of 2π‐CSAP‐s. As indicated above, in SLOW‐editing, two different 2π‐CSAP‐pairs that differ in their refocusing‐bandwidth Δω_RF_ are used: the first pair refocuses all resonances of the J‐coupled spin system under investigation, whereas the second pair refocuses only a part of the coupled spin‐system resonances. Figure 4A shows the simulation results of the adiabatic refocusing pulses with varying BW and carrier frequency used in three different editing schemes. The proposed schemes result in J‐difference spectra of 2HG_1.88_ (meaning the 2HG signal at 1.88 ppm), 2HG_4.01_, GABA_3.00_, Glx_3.77_ (glutamate and glutamine), PE_3.22_, and Glx_2.11_. The inserted figure of scheme 2 and 3 illustrates the finer scale of the pulse inversion profile. It should be mentioned that, although the inversion profile (for instance scheme 2) is close to 80% (i.e., M_z_ ∼ −0.8) at 4.2 ppm, the corresponding refocusing profile can archive more than 90% (Supporting Information Figure S16B). In addition, please note that the scheme 1 only illustrates the SLOW working principle and has not been applied in vivo.

In Figure 4B, the in vitro measurements 2HG‐editing (scheme 1), GABA‐editing (scheme 2), and PE‐editing (scheme 3) are shown. For instance, in SLOW GABA editing, the first measurement (indicated in blue) refocuses the whole GABA spectrum in the 1.6–4.2 ppm range (referred to by editing‐full). In contrast, the second measurement (displayed in orange) is obtained by refocusing only the 2.7–4.2 ppm range (editing‐partial), thus refocusing only the multiplet around 3.0 ppm. Like MEGA‐editing, SLOW‐editing also requires subtraction of the two responses to obtain the edited spectrum (shown in purple).

In Figure 4C, the J‐difference^28^ simulations for metabolites are performed using in‐house MATLAB‐code with above‐mentioned SLOW‐editing schemes. The in vitro editing results agree with the corresponding simulations (Figure 4B,C).

#### 3.2.2 In vivo 2HG SLOW‐editing

**FIGURE 5:**
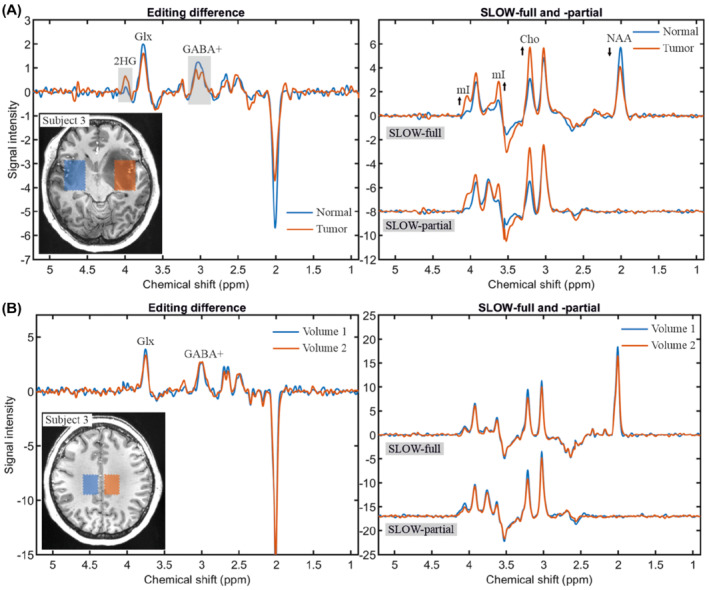
In vivo measurement of 2HG and GABA+ using SLOW‐editing scheme 2. A, The editing difference, SLOW‐full and ‐partial in the normal (blue) and tumor (orange) tissues. The selected volumes (30.1 × 38.7 × 7.8 mm, 7 × 9 × 1 = 63 voxels) are indicated on the left T1‐weighted MRI. B, The editing difference, SLOW‐full and ‐partial in the left normal (blue) and right normal (orange) tissues of the same subject, but at different localization. The selected volumes (21.5 × 30.1 × 7.8 mm, 5 × 7 × 1 = 35 voxels) are indicated on the left T1‐weighted MRI. TE = 68 ms, TR = 1500 ms, matrix = 65 × 23 × 9 (4.3 × 7.8 × 7.8 mm), and measurement time = 9:04 min

Figure 5 shows the result obtained with SLOW‐editing (scheme 2) to detect 2HG_4.01_ in a histologically confirmed IDH1‐mutated^5^, ^9^, ^10^, ^29^, ^30^, ^31^, ^32^, ^33^ glioma‐patient. The presence of 2HG in the tumor compared with the contralateral normal tissue is clearly identifiable (Figure 5A). In addition, the observed decrease of N‐acetyl aspartate (NAA) and the increase of choline (Cho) as well as myo‐inositol (mI) is also typical in IDH1‐mutated gliomas. The co‐edited GABA and glutamine and glutamate (Glx) spin systems are seen both in tumor and normal tissue (Figure 5A,B), which are slightly lower in the lesion. The spectra of two anatomic mirror‐symmetric normal tissue samples show, highly identical spectral profiles (Figure 5B).

#### 3.2.3 In vivo GABA+ and PE SLOW‐editing

**FIGURE 6:**
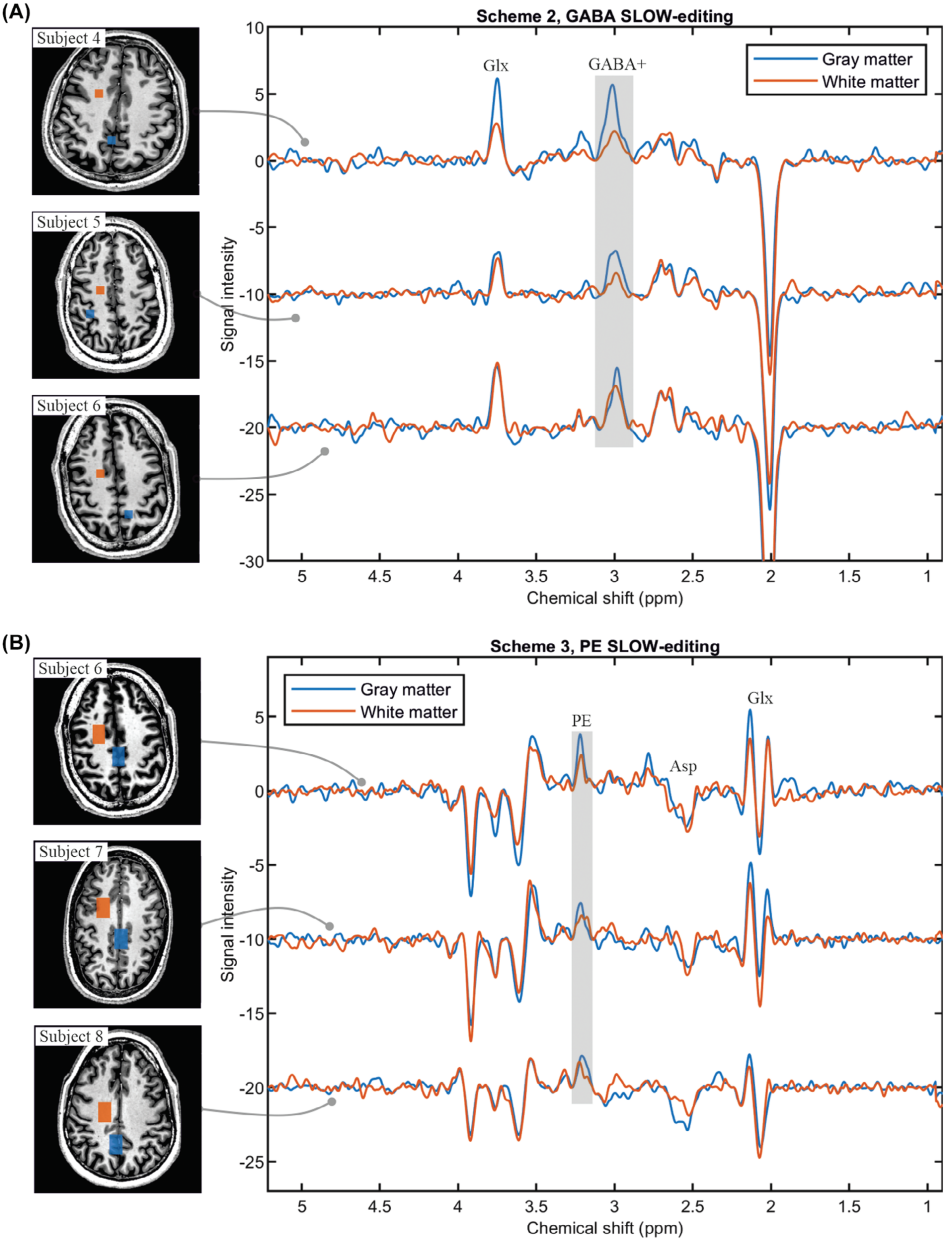
In vivo measurement of GABA+ and PE using SLOW‐editing scheme 2 and 3. A, GABA+‐editing using editing scheme 2 (TE = 68 ms) in three healthy volunteers, the selected volumes (8.6 × 8.6 × 7.8 mm, 2 × 2 × 1 = 4 voxels) are indicated on the left T1‐weighted MRI. The gray and white matter are marked as blue and orange, respectively. B, PE‐editing using scheme 3 (TE = 90 ms) in three healthy volunteers, the selected volumes (12.9 × 21.5 × 7.8 mm, 3 × 5 × 1 = 15 voxels) are indicated on the left T1‐weighted MRI. The gray and white matter are marked as blue and orange, respectively. TR = 1500 ms, data matrix = 65 × 23 × 9 (4.3 × 7.8 × 7.8 mm), and measurement time = 9:04 min

**FIGURE 7:**
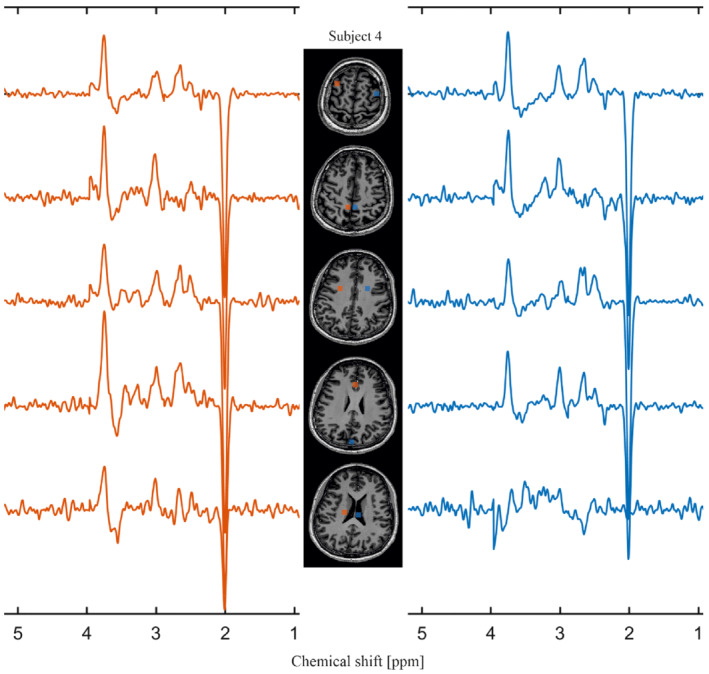
In vivo measurement of GABA+ using scheme 2 with multiple slices on subject 4. The selected volumes (8.6 × 8.6 × 7.8 mm, 2 × 2 × 1 = 4 voxels) are marked on the T1‐weighted MRI in the center. TR = 1500 ms, data matrix = 65 × 23 × 9 (4.3 × 7.8 × 7.8 mm), and measurement time = 9:04 min

SLOW‐editing scheme 2 and 3 were performed on three healthy subjects, respectively (Figure 6). GABA+ refers to GABA and co‐edited macromolecule which has J‐coupling resonances at 1.7 and 3 ppm.^8^ The selected volumes are in gray matter (blue) and in white matter (orange) as indicated on the T1‐weighted MRI. It is obvious that the level of GABA+ is higher in gray matter than in white matter (Figure 6A), whereas the difference of PE level is not as obvious as that of GABA+ (Figure 6B). The co‐edited Glx around 2.11 ppm is also seen in PE SLOW‐editing, and its spectral patterns match the simulated spectral pattern closely. In addition, Figure 7 shows GABA+ editing results of one subject for several selected volumes within multiple slices. Supporting Information Table S2 of the Supporting Information gives typical numeric values on the Cramér‐Rao bounds for SLOW‐full, SLOW‐partial, and SLOW‐difference editing spectra.

## 4 DISCUSSION

### 4.1 Chemical‐shift selective adiabatic refocusing

#### 4.1.1 *B*_1_ ^+^‐inhomogeneities

Our 2π‐CSAP‐EPSI offers a robust way to sufficiently tackle *B*_1_^+^ inhomogeneities problem inherent to UHF‐MRS(I). We could show (Figure 3E) that the 2π‐CSAP uniformly refocuses the spins over the complete in vivo volume of interest, including the deeper located brain regions in the center of the measurement volume. This excellent in‐plane refocusing/editing performance could not be reached by (MEGA‐)semiLASER over the same volume because (a) the necessary peak power to keep the CSDA within acceptable bounds is not available in our 7T‐Terra system; and (b) even when using peak‐power minimized adiabatic RF‐refocusing pulses, the TR had still to be increased due to remaining SAR limitations, which implied clinically unacceptable total acquisition time; details given below.

#### 4.1.2 SAR and RF peak power considerations

In contrast to amplitude modulated pulses (e.g., *Mao* or sinc pulses) the required peak power of adiabatic RF pulses is to some extend decoupled from the RF‐bandwidth Δω_RF_ and can be reduced in theory by increasing pulse duration_._ This means that a long duration adiabatic pulse can substantially reduce required RF peak power.

Therefore, the pulse duration of the two slice‐selective adiabatic 2π‐pulse pairs (2π‐SSAP) cannot be chosen short enough to fulfill both SAR/peak‐power constraints with MEGA‐semiLASER and optimal editing TE requirements. As a result, the effective realizable TE was slightly longer than the optimal 1/2 J duration (68 ms for GABA) on our clinical 7T MR scanner.

In contrast, under clinically relevant conditions, 2π‐CSAP/SLOW‐EPSI is not restricted by SAR and limited RF peak power whatsoever, because of the narrow BW and long duration of refocusing/editing adiabatic pulses. The low SAR (7.6%) and RF peak power (15.7%) enables short TR (1.5 s) for our method to scan the whole‐brain within a clinically acceptable measurement time. In contrast, long TR (≥ 4.5 s) in MEGA‐semiLASER^16^ must be chosen, resulting in unacceptable long measurement times when applied in vivo. In SLOW‐editing, this leaves available SAR to be used for additional more sophisticated chemical‐shift selective lipid suppression in the preparation phase of the pulse sequence, as was done in this study. The details are described in the Methods section.

On theoretical considerations, the expected SAR and peak‐power advantages of SLOW‐editing compared to MEGA‐semiLASER editing become greater when the magnetic field strength exceeds 7T. However, whether SLOW‐editing applied at *B*_0_ > 7T as proposed here will still work satisfactorily in CP mode needs further investigation.

#### 4.1.3 CSDA considerations

Whereas MEGA‐semiLASER based MRSI at UHF suffers from severe CSDA in both the slab selection *and* both in‐plane directions, SLOW‐editing only suffers from the CSDA artifacts in the slab selection direction. This makes the spatial resolved data obtained SLOW‐editing substantially easier to interpret, since incomplete refocusing and editing performance are only present in the outer slices of MRSI‐slice stack. In 3D‐MEGA‐semiLASER MRSI‐based editing, the CSDA also severely affects the in‐plane spectroscopic images, becoming more and more severe with increasing excited volume of interest.

#### 4.1.4 Signal foldback considerations

Since the spectral width required to cover all metabolite of interest scales with *B*_0_, larger spectral bandwidths are required at UHF. For accelerated MRSI, such as EPSI,^20^ radial‐EPSI,^34^ and spiral^35^ type of readout schemes, this means that the gradient slew rates can become the limiting factor to prevent aliasing artifacts. However, the 2π‐CSAP in SLOW‐editing would not rephase the metabolite‐signals from the outside of acquisition sweep‐width range (∼0.8–5.2 ppm), thereby avoiding aliasing artifacts. This is very easy to realize using 2π‐CSAP but much harder to realize using 2π‐SSAP as in semiLASER. For this reason, the spectra obtained with our proposed 2π‐CSAP show substantially less baseline‐role effects.

#### 4.1.5 Lipid and water suppression

The use of 2π‐CSAP has an implicit additional excellent homogeneous water and fat suppression obtained at *zero* SAR cost. The suppression factors of these signals improve with increasing 2π‐CSAP pulse duration *T*_2π_. This is because the width of transition band (Δω_TB_) of RF pulses is inverse proportional to their duration (Δω_TB_ ∝1/*T*_2π_). For maximal lipid/water suppression, the transition band should be as narrow as possible, which favors long pulse duration *T*_2π_. In the case of 2π‐CSAP‐EPSI, the unwanted signals (>4.6 ppm and <1.4 ppm) are almost perfectly dephased (suppressed), often resulting in excellent flat baselines spectra. Again, this is difficult to obtain in MEGA‐semiLASER based acquisition schemes (see previous paragraph).

#### 4.1.6 Influence of *B*_0_ shimming

In UHF, *B*_0_ shimming is challenging and can have a major impact on the ability to be able to quantify the spectra. Apart from line broadening, which elevates the Cramer‐Rao minimum‐variance‐bound values (CR‐MVB),^36^, ^37^ also remote lipid signals can fold into the 1.8–4.5 ppm range.

Because SLOW does not use in‐plane spatial localization, some lipid signals in voxels near the skull may shift into the 1.8–4.5 ppm range, even when using up to third‐order shim coils in the 7T Terra system. These lipid‐signals emerge from frequency shifted lipid resonances (due to imperfect B_0_ shimming), which are partially in the pass and transition band of the 2π‐CSAP. However, the signals can be handled by post‐processing.^38^, ^39^

#### 4.1.7 Spectral quality, SNR, and metabolite mapping

Since the 7T Terra system has up to third‐order shimming coils, the shimming is sufficiently good in approximately 70%–80% of the brain. Shimming is problematic in areas right above the nasal cavity and lower parts of the brain. To demonstrate the spectral quality that can be obtained with SLOW‐EPSI, Supporting Information Figure S12 displays a matrix of spectra of single voxels from a representative dataset and the SNR of GABA+ for each individual voxel. For GABA+, a single voxel SNR of approximately 2 can be reached.

Supporting Information Figures S13 and 14 show metabolite maps and SNR of Cr+ (Cr and GABA+), Cho, GABA+, and Glx obtained by Gaussian fitting. The post‐processing was described in Supporting Information .

#### 4.1.8 Acquisition time

The measurement time for the displayed 3D‐resolved example was about 8 min *without* parallel imaging techniques. This means that even a shorter scan times could be achieved by using GRAPPA^40^ or SENSE.^41^ In the UHF‐MRSI area, in addition to a robust *B*_1_^+^/*B*_0_ inhomogenieties, it is essential to achieve a good compromise between measurement time, SNR, and SAR. Modern scanners, meanwhile, have various techniques for parallel imaging and k‐space sampling that could be made usable in this context. However, it must be considered that GRAPPA is associated with a loss of SNR and methods such as simultaneous multi‐slice^42^ or simultaneous echo^43^, ^44^ or image refocusing^45^ result in a modification of the source code of the sequence. For non‐edited EPSI datasets this SNR loss could be acceptable; however, whether for spectral editing the SNR would still be sufficient must be investigated.

### 4.2 Spectral editing performance

In MEGA‐semiLASER‐based editing, broadband 2π‐SSAP and narrow band AM‐modulated Gaussian pulses are used for refocusing and editing,^8^, ^16^ which imply the above‐mentioned difficulties and have a severe impact on the editing performance in MRSI and severely limits clinical applicability, especially at UHF. In contrast, the advantages of using 2π‐CSAP are fully used in SLOW‐editing combined with MRSI; and include (1) robustness to *B*_1_^+^‐homogeneous refocusing, even in clinical CP‐mode on the 7T‐Terra system using a Nova head‐coil) and therefore yield a spatial homogeneous editing efficiency; (2) low SAR; (3) low RF peak power; (4) no in‐plane CSDA; and (5) no foldback aliasing artifacts, and superior implicit water and lipid suppression obtained at zero SAR cost.

#### 4.2.1 Refocusing and editing pulses

In MEGA‐editing, refocusing and editing is obtained by separate pulses, whereas in SLOW‐editing they are identical. This not only extremely simplifies SLOW‐editing sequences (only two RF pulses necessary) compared to MEGA, but also minimizes the effects of non‐ideal RF‐pulse behavior. Apart from the fact that 2π‐CSAP significantly reduces SAR, it could be further reduced by applying SLOW‐full/partial in an interleaved way. Since the Δω_RF_[SLOW‐partial] < Δω_RF_[SLOW‐full], their associated SAR loads are SAR[SLOW‐partial] < SAR[SLOW‐full]. Therefore, the interleaved version of SLOW‐full/‐partial will reduce the average SAR even further.

#### 4.2.2 B_0_ inhomogeneities and editing performance

Due to the larger influence of susceptibility differences at UHF, the *B*_0_ inhomogeneities to cope with are more pronounced, especially when targeting the whole brain. Even the best *B*_0_ shimming using up to third‐order shimming still results in residual *B*_0_ inhomogeneities ≥0.1 ppm. Together with *B*_1_^+^ inhomogeneities, these factors have a significant negative effect on the editing accuracy using narrow‐band Gaussian‐shaped editing pulse. The two reasons for this are that (a) the pulse flip angle of the amplitude modulated Gaussian‐editing pulses are very sensitive to both *B*_1_^+^ inhomogeneity since they are non‐adiabatic, and (b) the offset frequency variations result in additional pulse flip‐angle reduction due to the narrow RF‐BW combined with the wide transition band inherent to Gaussian‐modulated RF‐pulse shapes. The editing performance in this situation is highly spatial dependent, making the analysis of in vivo data very difficult, if not impossible. This in contrast to SLOW‐editing, which uses highly frequency‐selective 2π‐CSAP that are extremely robust to both *B*_0_ and *B*_1_^+^ inhomogeneities.

The fact that SLOW operates with extremely sharp transition bands does not imply that SLOW would be susceptible to B_0_ inhomogeneities. To make this clear, for instance, in scheme 2 (GABA+ editing), the passband of editing‐full starts from 1.6 ppm (Supporting Information Figure S16), which is 0.3 ppm away from GABA at 1.9 ppm. For the editing‐partial, the passband starts at 2.7 ppm, which is also 0.3 ppm away from GABA at 3.0 ppm. In addition, the stop band begins at 2.35 ppm, which is 0.45 ppm away from GABA at 1.9 ppm. Given this, application of SLOW for GABA+ editing is robust to ΔB_0_ < ± 0.3 ppm. In contrast to “classical” MEGA, using narrow band Gaussian‐shaped MEGA pulses, the resonance frequency must be within a ΔB_0_ < ±0.05 ppm (full width at 95% maximum) exactly,^18^ which directly influences the editing efficiency. In MEGA using adiabatic editing pulses^18^ (robust to ΔB_0_ < ± 0.15 ppm as reported), the situation is better than classical MEGA, but still worse than SLOW. However, MEGA using adiabatic editing pulses may achieve better B_0_ robustness than reported, by fine tuning the carrier frequency of the MEGA‐pulses. Please note that the safety margin of 0.3 ppm applies to GABA+ editing only (scheme 2), whereas a safety margin of 0.2 ppm is valid for 2HG (scheme 2) and PE (scheme 3) (see Figure 4 and Supporting Information Figure S16).

#### 4.2.3 Water/lipid suppression limitation

Because of the identical refocusing pulse bandwidth range in MEGA‐on and ‐off, additional residual water/lipid suppression is obtained by subtraction of the datasets. In contrast, SLOW‐editing does not have this advantage, due to different bandwidth ranges of the full and partial pulses used. Practically, however, the observed residual water signals in SLOW‐EPSI are ignorable and never posed a problem in vivo. The remaining strong lipids signals appear in the voxels near the skull (see Supporting Information Figures S10 and S11), and can be removed by post‐processing techniques, like for example, references.^38^, ^39^

#### 4.2.4 Lactate limitation

The lactate at 1.31 ppm is also suppressed by the 2π‐CSAP pulse just like lipid, therefore the lactate signal at 1.31 ppm cannot be detected. This issue also happens to similar whole‐brain fast MRSI sequence^18^, ^25^ due to the need to suppress lipid signals. However, our 2π‐CSAP (for example, SLOW scheme 2) only refocuses the 4.1 ppm quartet of lactate and dephases the spins at 1.31 ppm, so the J‐coupling of 4.1 ppm quartet is fully refocused. Hence, the 2π‐CSAP/SLOW‐editing gain signal intensity for quartet of lactate, which means that lactate can still be quantified by fitting.

#### 4.2.5 TE limitation

The shorter the 2π‐CSAP pulse durations are, the broader the transition‐bands become, resulting in poorer frequency selectivity, and less good water and lipid suppression, which is a limitation 2π‐CSAP refocusing. Nevertheless, a minimal‐TE of 30 ms (including spoiler gradient duration) can be reached on a 7T system while still having sufficient water suppression, but substantially less lipid, suppression. Additionally, this shorter TE‐times results in a slight loss of metabolite‐signals close to the transition band; for instance, for spins in the 3.7–4.0 ppm offset range. It should be noted that the mentioned TE limitation of 30 ms at 7T applies to 2π‐CSAP‐EPSI only, and not to SLOW‐EPSI. This is because the optimal J‐difference editing TE of the major brain metabolites is at least 68 ms.

#### 4.2.6 Comparison of SLOW‐EPSI to other UHF MRSI methods

There are several spin‐echo based MRSI sequences using adiabatic pulses at UHF for instance.^18^, ^25^, ^46^ However, none of these methods allow for whole‐brain MRSI *and* spectral editing. Reference 18 used adiabatic MEGA‐editing pulses together with one pair of GOIA‐W(16,4) refocusing pulses^23^, ^47^ (8 ms duration, and 10 kHz BW with a B_1_^+^ security range of 40% above the adiabatic threshold). The SLOW‐editing, for instance scheme 2, used 2π‐CSAP (complex secant hyperbolic shape) with 24 ms duration, 0.56 (partial) – 0.88 (full) kHz, and 100% above the adiabatic threshold B_1_^+^ (Supporting Information Figures S8 and S9). Furthermore, assuming the same adiabatic condition, the SAR of our 2π‐CSAP based method (editing‐full), is 1–0.88/10 = 91% lower than in Reference 18. Moreover, the method of Reference 18 is a single slice method, with TA = 24:12 min, whereas SLOW‐EPSI is whole brain and has TA = 9:04 min.

In Reference 25, a whole‐brain MRSI method (GOIA‐W(16,4) for refocusing, 5 ms duration, and 20 kHz BW, with a B_1_^+^ security range of 20% above the adiabatic threshold) is presented without spectral editing. It is reported to have a TR = 1.8 s, operating at SAR‐levels between 60% and 95% of the maximal allowed SAR, and TA of 11:38 min. SLOW‐editing could be implemented in this sequence at the expense of more SAR, and a TA of 23:16 min, whereas whole‐brain SLOW‐EPSI only requires TA of 9:04 min and is 100% above the adiabatic threshold and still not reaching the SAR limitation. The method uses temporal interleaves for read‐out, which SLOW does not need, therefore, gainimg SNR. However, the CSDA of Reference 25 is lower and the reported voxel‐size is lower than SLOW‐EPSI.

In Reference 46 a single UHF‐MRSI SENSE‐based method without outer volume suppression was proposed which is single slice and does not include spectral editing. The authors report a TA of 12:27 s at a SAR level of just 53%, a TR of 4.5 s having spectroscopic readout. Also, this method could be extended with a SLOW‐editing option by replacement of their SSAP with CSAP. However, the total measurement will be considerably longer than SLOW‐EPSI.

#### 4.2.7 Clinical applicability

At UHF, our general results obtained in clinical cases (not shown here) strongly suggest that both the 2π‐CSAP‐EPSI and the SLOW‐editing technique have huge potential for clinical neuro‐spectroscopy applied to clinical neuro oncology (2HG) and mental and neurodegenerative diseases (GABA). At 7T, the proposed whole‐brain MRSI sequence requires less than 5 min for recording non edited spectra (2π‐CSAP‐EPSI), and 10 min to perform editing (SLOW‐EPSI).

In summary, the presented SLOW‐editing technique provides substantial advantages over the classic MEGA‐semiLASER technique^16^ with spectral read‐out at UHF with respect to: (1) ease of use, (2) very low SAR and RF peak‐power burden, (3) implicit additional water/lipid‐suppression at zero SAR‐cost, and (4) spatial homogeneous editing‐performance enabling unambiguousness interpretation of the data.

Furthermore, compared to MEGA 1D‐semiLASER,^18^ this method has also a spatial homogeneous editing performance like SLOW‐editing. However SLOW‐editing allows for substantially shorter TR (namely 1.5 instead 2.8 s), is whole brain, and the use of 2π‐CSAP minimizes spectral aliasing, resulting in minimal artifacts and baseline distortions, which allows narrow acquisition spectral BW (∼0.8–5.2 ppm). SLOW‐editing was successfully tested (in vitro and in vivo) at 7T in more than 10 brain tumor patients, and 20 healthy subjects. Finally, it was shown that, alike MEGA, SLOW‐editing can be used, for main important hidden brain‐metabolites which are 2HG, GABA/GABA+, PE, and Glx.

## CONFLICT OF INTEREST

We disclose that the application of chemical‐shift selective adiabatic 2π‐pulse (CSAP) and SLOW‐editing described in the paper has been filed with the European Patent Office in The Hague (The Netherlands) awaiting the decision. Patent applicant: Universität Bern. Name of inventors: Guodong Weng and Johannes Slotboom (equal shares and contributions). Application number: EP21171729.3.

## Supporting information


**Figure S1.** Sequence‐scheme in Siemens IDEA VE12U‐platform.
**Figure S2.** Asymmetric adiabatic inversion recovery lipid suppression pulse.
**Figure S3.** In vitro measurement of SLOW‐editing with asymmetric adiabatic inversion recovery lipid suppression pulse.
**Figure S4.** Phantoms.
**Figure S5.** Flowchart of reconstruction and pre‐post‐processing.
**Figure S6.** The user interface (UI) of spectrIm‐QMRS.
**Figure S7.** Simulation of the metabolite spectrum basis (Editing scheme 2, SLOW‐full).
**Figure S8.** In vitro Cr integration map using SLOW‐partial (scheme 2) with different B1 amplitudes of 2π‐CSAP.
**Figure S9.** In vivo SLOW‐partial (scheme 2) with different B1 amplitudes of 2π‐CSAP.
**Figure S10.** SLOW‐EPSI (scheme 2) of a healthy subject (#9)
**Figure S11.** SLOW‐EPSI (scheme 2) of a healthy subject (#9).
**Figure S12.** GABA+ editing (scheme 2) of a healthy subject (#9).
**Figure S13.** Cr+ and Cho maps of a healthy subject (#9).
**Figure S14.** Glx and GABA+ maps of a healthy subject (#9).
**Figure S15.** Spectral fitting.
**Figure S16.** Simulation for complex secant hyperbolic adiabatic pulse (scheme 2, editing‐full).
**Figure S17.** Simulation for WURST‐16 adiabatic pulse.
**Figure S18.** In vitro Cr integration map with different B1 amplitudes of 2π‐CSAP (TE 30 ms).
**Table S1.** The RF‐pulse parameters of adiabatic pulses used.
**Table S2.** CR‐MVB Relative %‐errors.
