# Supplementary material for: SLOW: A novel spectral editing method for whole‐brain MRSI at ultra high magnetic field
Source: Magn Reson Med. 2022 Mar 28;88(1):53–70. doi: 10.1002/mrm.29220 (PMC9212787; doi:10.1002/mrm.29220)
Supplement: Supplementary file 1 — Figure S1. Sequence‐scheme in Siemens IDEA VE12U‐platform. Figure S2. Asymmetric adiabatic inversion recovery lipid suppression pulse. Figure S3. In vitro measurement of SLOW‐editing with asymmetric adiabatic inversion recovery lipid suppression pulse. Figure S4. Phantoms. Figure S5. Flowchart of reconstruction and pre‐post‐processing. Figure S6. The user interface (UI) of spectrIm‐QMRS. Figure S7. Simulation of the metabolite spectrum basis (Editing scheme 2, SLOW‐full). Figure S8. In vitro Cr integration map using SLOW‐partial (scheme 2) with different B1 amplitudes of 2π‐CSAP. Figure S9. In vivo SLOW‐partial (scheme 2) with different B1 amplitudes of 2π‐CSAP. Figure S10. SLOW‐EPSI (scheme 2) of a healthy subject (#9) Figure S11. SLOW‐EPSI (scheme 2) of a healthy subject (#9). Figure S12. GABA+ editing (scheme 2) of a healthy subject (#9). Figure S13. Cr+ and Cho maps of a healthy subject (#9). Figure S14. Glx and GABA+ maps of a healthy subject (#9). Figure S15. Spectral fitting. Figure S16. Simulation for complex secant hyperbolic adiabatic pulse (scheme 2, editing‐full). Figure S17. Simulation for WURST‐16 adiabatic pulse. Figure S18. In vitro Cr integration map with different B1 amplitudes of 2π‐CSAP (TE 30 ms). Table S1. The RF‐pulse parameters of adiabatic pulses used. Table S2. CR‐MVB Relative %‐errors. [file MRM-88-53-s001.docx]

## Supporting Information

## SLOW: a novel spectral editing method for whole-brain MRSI

## at Ultra High Magnetic Field

## IR lipid suppression pulse

The pulse shape and inversion profile are shown on Supporting Information Figure S2, and pulse parameters are listed on Supporting Information Table S1. The bandwidth for the sharp transition band on the left side is 33 Hz (0.11 ppm). The full stop of transition band at 1.89 ppm is 0.12 ppm away from NAA (2.01 ppm). The goal is to suppress lipid residues as much as possible without affecting NAA in most regions, for example SLOW-editing scheme 2, to obtain a flat baseline for editing resonance signal by subtracting SLOW-full and -partial. For non-editing 2π-CSAP, one may consider setting the IR pulse further away NAA, for instance 0.15 – 0.2 ppm, to ensure that the NAA resonance remains unaffected in even larger regions.

Supporting Information Figure S3 shows that there is no influence on the editing result of 2HG_4.01_ when suppressing 2HG_1.83_ using IR pulse.

## Reconstruction and pre-post-processing

The flowchart of reconstruction and pre-post-processing is illustrated on Supporting Information Figure S3.

### *Raw data*

The measured raw-data is reconstructed using ICE and stored into DICOM format, which is further processed by Metabolic Imaging Data Analysis System (MIDAS)^1^.

### *MIDAS*

MIDAS is a software-program written in IDL™ (Harris Geospatial Solutions, Inc., Colorado, USA) used to process EPSI-data. The processing-pipeline is the following:

1. Non-uniform EPSI readout k-space regridding is accomplished using interlaced Fourier-transform^2^.
2. The even and odd echoes are averaged with echo-drift-correction.
3. Spatial Fourier-transforms (FFT) for water reference data.
4. Linear regression was performed for FID points 3 and 7 to create phase and magnitude correction functions using water reference data. The reason for this is to avoid oscillations in the 1st few echoes. Then a weighted combination was performed for multi-channels^3^. The phase and magnitude correction and weighting of water reference data were saved and then used in the subsequent spatial FFT of metabolite data.
5. Spatial Gaussian filter and Spectral FFT-with Gaussian-filter (with a broadening-parameter in the range of 2 - 6 ).

### *spectrIm-QMRS or MATLAB*

The desktop application spectrIm-QMRS is an in-house developed software package for *q*uantitative *m*agnetic *r*esonance *s*pectroscopy (QMRS) is shown in Supporting Information Figure S4.

1. Automatic phase-correction and frequency-shift correction was applied based on the creatine/choline-resonance at 3ppm.
2. A 2D- or 3D-moving-median/average filter in time-domain can be applied.
3. For spectral-editing, the subtraction of editing-full minus editing-partial signal was applied.
4. Apodization-filter in time-domain.
5. Baseline-correction, lipid contamination and residual water (in very rare cases) removal using splines.

### *Spectral fitting*

The spectral-fitting could also be done in spectrIm-QMRS using the simulated metabolite spectrum basis generated with the MATLAB-code developed in-house. The example of the simulated spectral basis for SLOW-full (editing scheme 2) is shown in Supporting Information Figure S7. And an example of fitting is shown in Supplementary Information Figure S15 and Table S2.

The fitting program TDFDFit^4^ is, a parameters least-squares fitting-method, based on fitting using metabolite basis sets to fit the area, phase and frequency offset, as well as Lorentzian and Gaussian line-broadening model parameters. TDFDFit is included in package spectrIm-QMRS and can be downloaded from <http://spectrim.diskstation.me/spectrImWeb/>.

### *Mapping*

The mapping of Cr+ (3 ppm), Cho (3.2 ppm), Glx (3.75 ppm) and GABA+(3.01 ppm) was performed using a Gaussian fitting for each voxel. The B_1_ correction was applied using the map of water reference, that is:

$${Map}_{meta_{\_}corr}={Map}_{meta}/Map_{water}$$

### *Lipid suppression*

For scheme 2 editing-full, the transition band on the left hand side of the spectrum is between 4.2 ppm - 4.65 ppm, and for the right hand side of the spectrum between 1.60 ppm – 1.15 ppm. Due to the fact that lipid signals stem from areas are close to the receiver coil and are much harder to shim, lipid signal shifts in the range of 1.0 ppm – 2.5 ppm which leak into the voxels close to the skull (Supporting Information Figure S10 and S11).

The additional asymmetric adiabatic inversion recovery pulse was used to suppression shifted lipid signal in the range of 1.6 - 1.8 ppm, leaving the 1.8 – 2.5 ppm lipid contamination not handled. These lipid residual signals can, however, be further removed as described in literature references^5,6^.

For scheme 2 editing-partial, the transition band on the left hand side of the spectrum is between 4.2 - 4.65 ppm, and for the right hand side of the spectrum between 2.7– 2.35 ppm. In this case the lipid is as good as the water suppression with suppression factors larger than 1000s as displayed in the Supporting Information Figure S10 and S11.

## Adiabaticity

Supporting Information Figure S7 and S8 show the *in vitro* and *in vivo* measurement with different nominal B_1_ amplitudes (from 33.3% - 150%), with the mean and standard deviation indicated at the bottom left (S7). It is clear that the 2π-CSAP satisfies the condition of adiabaticity in the range of 50% - 150% of the nominal B_1_^+^ amplitudes.

In addition, the 2π-CSAP with short pulse time (8 ms and TE 30 ms) still satisfies the condition of adiabaticity in the range of 57% - 129% of the nominal B_1_^+^ amplitudes.

## Spectral quality, SNR, and metabolic mapping

### *Spectral quality and SNR*

To demonstrate the spectral quality and SNR of single voxels, Supporting Information Figure S12 shows a plot of voxels of the dataset. The noise level was calculated using the standard deviation of last 100 points on the right hand side of the spectrum (0.9 – 1.3 ppm). The signal level, for instance GABA+, was calculated using the mean value of GABA+ peak (40 points, 2.95 – 3.10 ppm).

### *Metabolite mapping*

Supporting Information Figure S11-S12 show the metabolite maps and SNR by Gaussian fitting of Cr+ (Cr and GABA+), Cho, Glx and GABA+ for multiple-slices of the dataset. The Cr+ and Cho maps were obtained using SLOW-partial dataset, and the Glx and GABA+ were obtained using SLOW-difference dataset (SLOW-partial minus SLOW-full).

## Reference

1. Maudsley AA, Darkazanli A, Alger JR, et al. Comprehensive processing, display and analysis forin vivo MR spectroscopic imaging. *NMR in Biomedicine*. 2006;19(4):492-503.

2. Bruder H, Fischer H, Reinfelder HE, Schmitt F. Image reconstruction for echo planar imaging with nonequidistantk-space sampling. *Magnetic Resonance in Medicine*. 1992;23(2):311-323.

3. Abdoli A, Maudsley AA. Phased-array combination for MR spectroscopic imaging using a water reference. *Magnetic resonance in medicine*. 2016;76(3):733-741.

4. Slotboom J, Boesch C, Kreis R. Versatile frequency domain fitting using time domain models and prior knowledge. *Magnetic Resonance in Medicine*. 1998;39(6):899-911.

5. Hu X, Levin DN, Lauterbur PC, Spraggins T. SLIM: Spectral localization by imaging. *Magnetic Resonance in Medicine*. 1988;8(3):314-322.

6. Kasten J, Klauser A, Lazeyras F, van de Ville D. Magnetic resonance spectroscopic imaging at superresolution: Overview and perspectives. *Journal of Magnetic Resonance*. 2016;263:193-208.

7. Andronesi OC, Ramadan S, Ratai EM, Jennings D, Mountford CE, Sorensen AG. Spectroscopic imaging with improved gradient modulated constant adiabaticity pulses on high-field clinical scanners. *Journal of Magnetic Resonance*. 2010;203(2):283-293.

8. Kupče Ē, Freeman R. Adiabatic Pulses for Wideband Inversion and Broadband Decoupling. *Journal of Magnetic Resonance, Series A*. 1995;115(2):273-276.


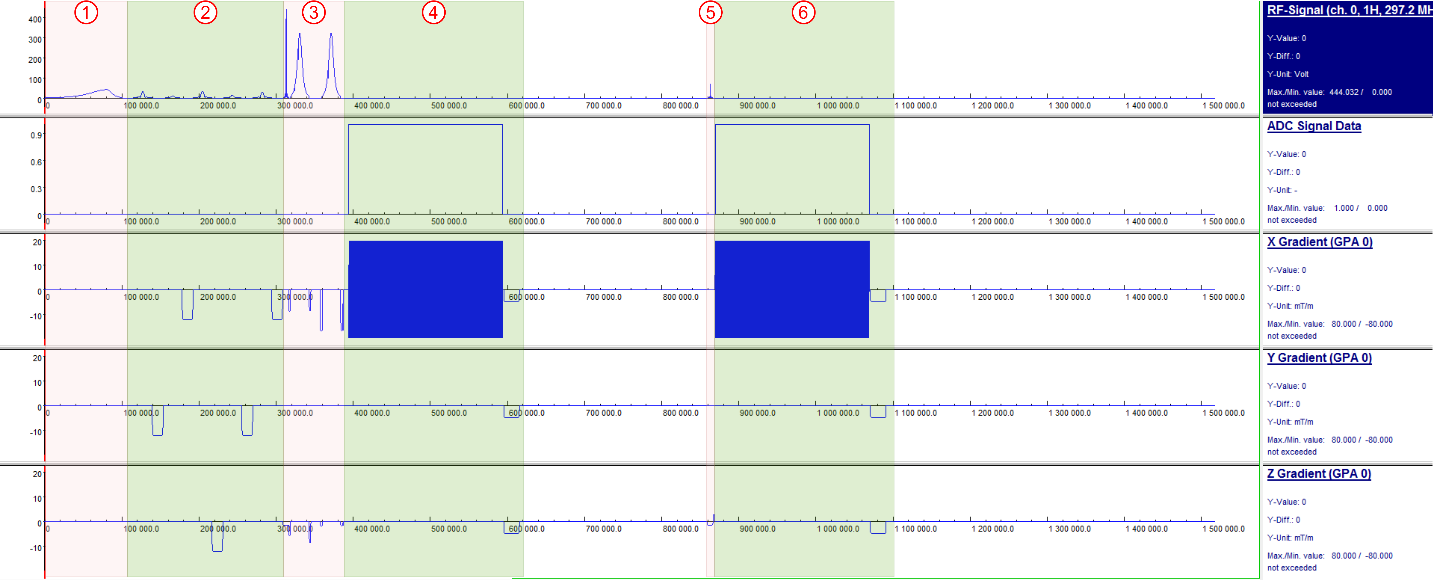


**Supporting Information Figure S1** **| Sequence scheme in Siemens IDEA VE12U platform. 1)** Asymmetric adiabatic inversion recovery lipid suppression pulse. **2)** Water suppression pulses. **3)** Slice selective excitation pulse and chemical selective adiabatic pulse pair. **4)** EPSI metabolite readout. **5)** Slice selective excitation pulse. **6)** EPSI water reference readout.


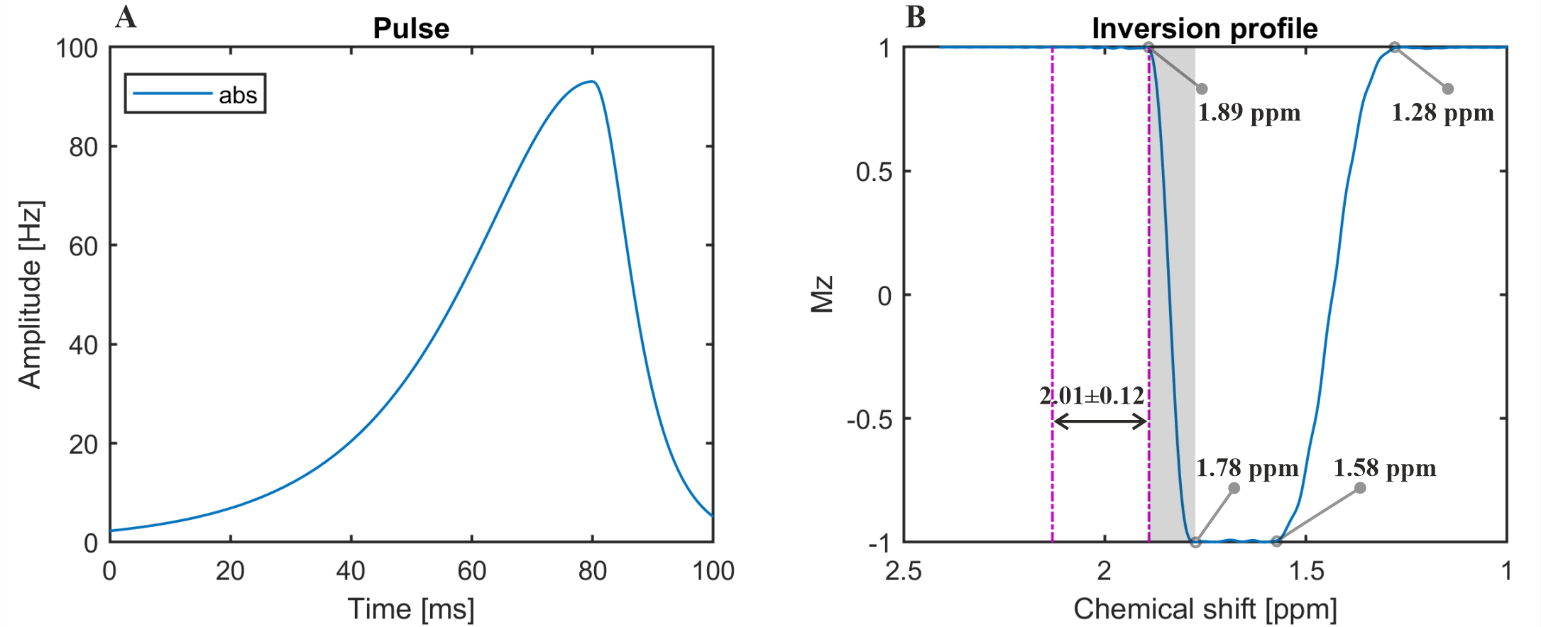


**Supporting Information Figure S2** **| Asymmetric adiabatic inversion recovery lipid suppression pulse. A)** The amplitude of the IR pulse. **B)** The inversion profile of the IR pulse. Transition bandwidth of the left hand side is 33 Hz (0.11 ppm) and is in grey.

**
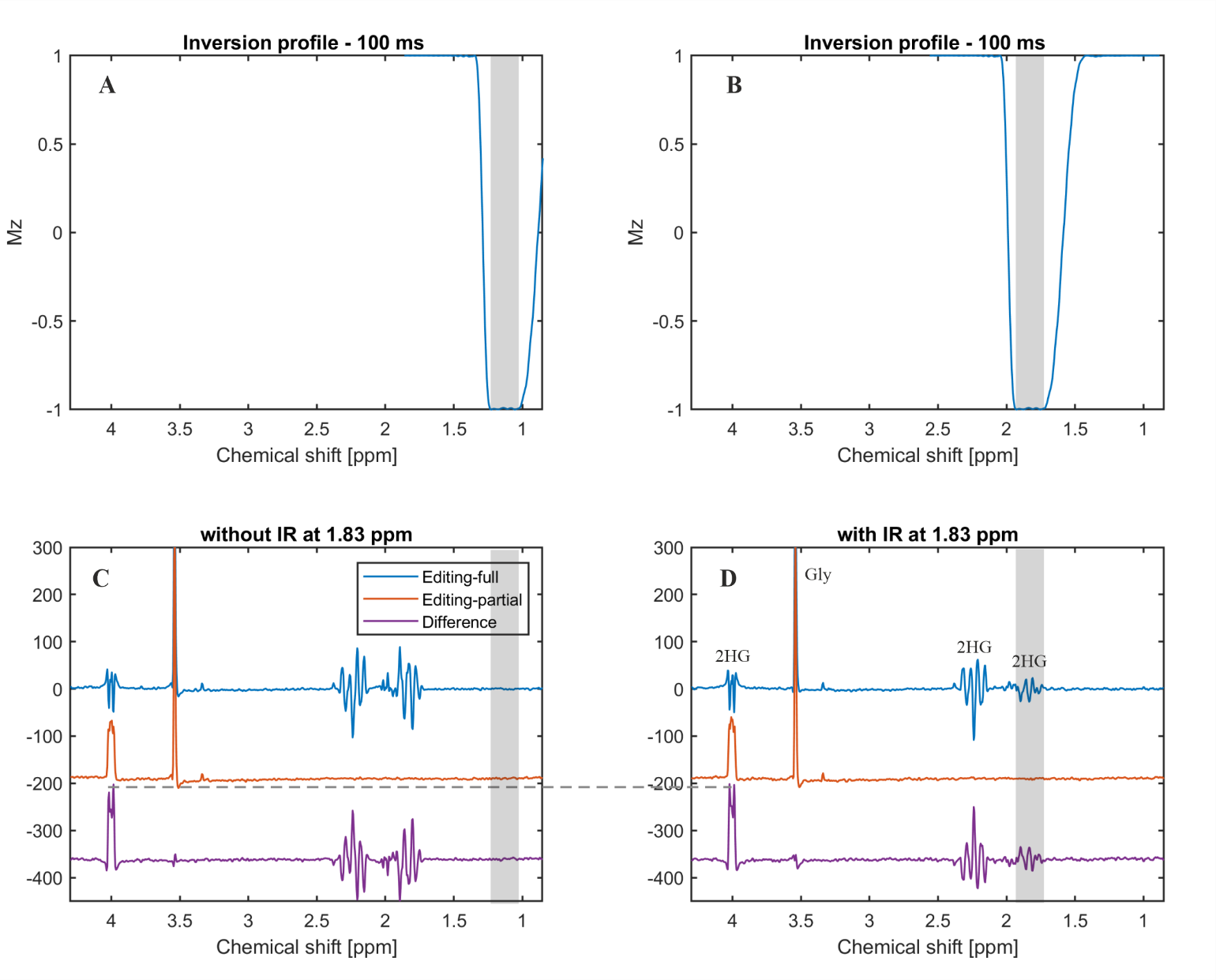
**

**Supporting Information Figure S3** **| In vitro measurement of SLOW-editing with Asymmetric adiabatic inversion recovery lipid suppression pulse. A-B)** The inversion profile of IR pulse without and with suppression 2HG_1.83_, respectively. **C-D)** Corresponding in vitro measurement with SLOW-editing scheme 2. TE = 68 ms, TR = 1500 ms, FOV = 280 × 180 × 60 mm, matrix = 65 × 23 × 5 (4.3 × 7.8 × 12 mm resolution), and TA = 2:26 min.


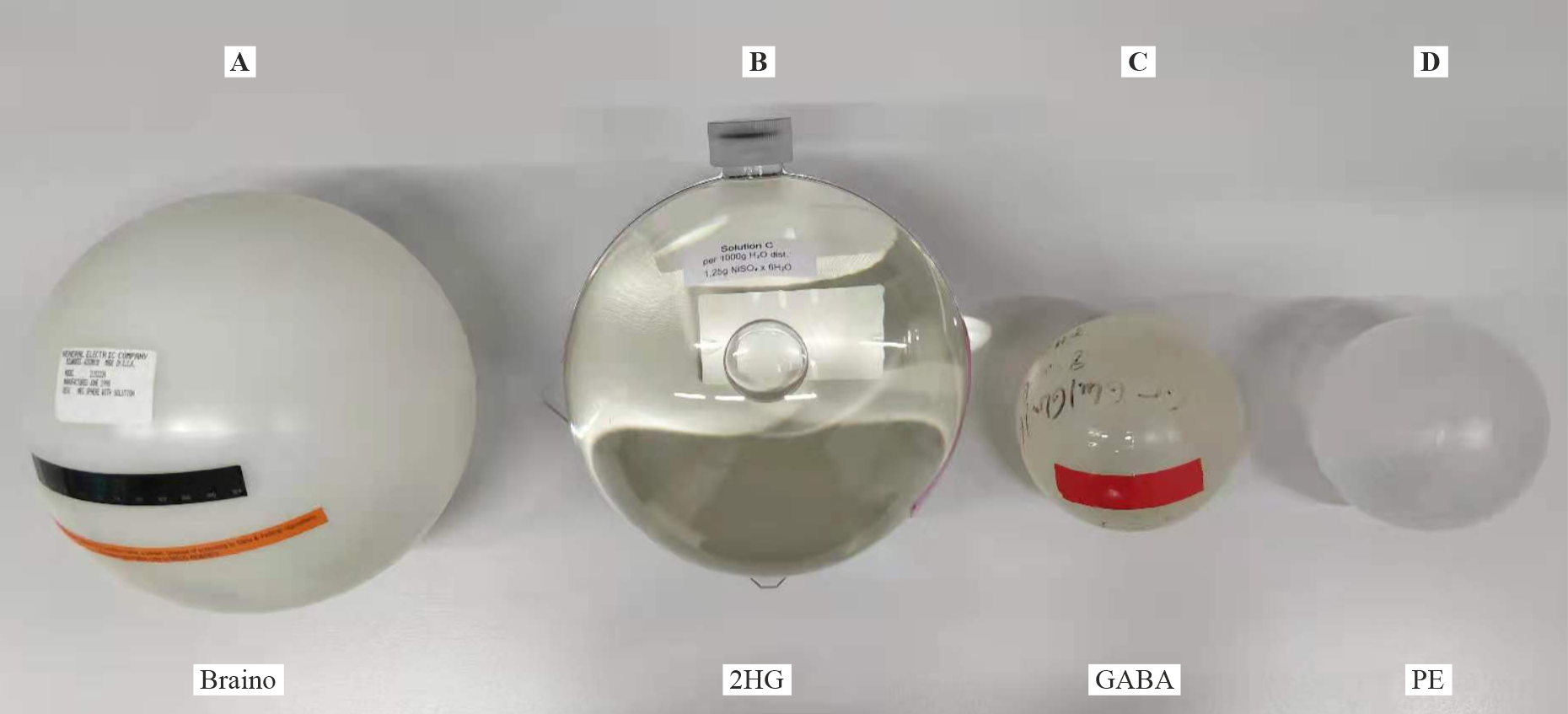


**Supporting Information Figure S4** **|** **Phantoms. (A),** Braino phantom (General Electric, USA). **(B),** 2HG phantom (~7.8 mmol/L of 2HG and 18 mmol/L of glycine). **(C),** GABA phantom house (~10 mmol/L of GABA, creatine, and glycine) . **(D),** PE phantom house (~14 mmol/L of PE)


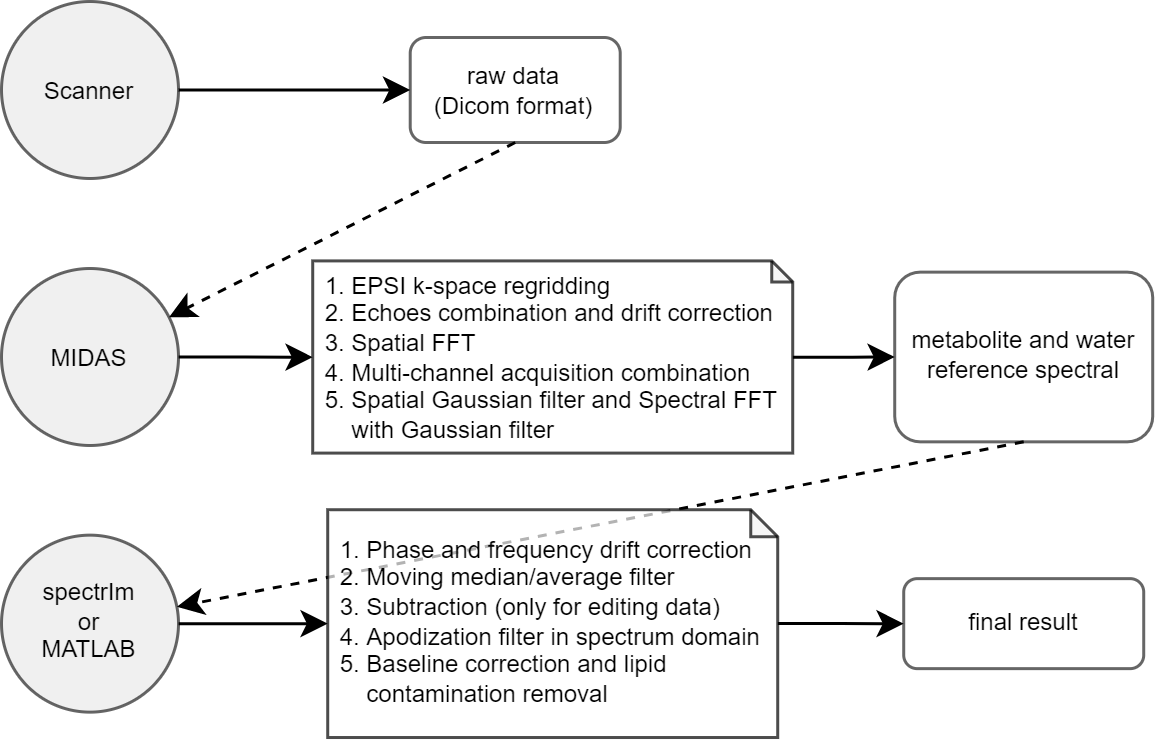


**Supporting Information Figure S5 | Flowchart of reconstruction and pre-post-processing.**

**
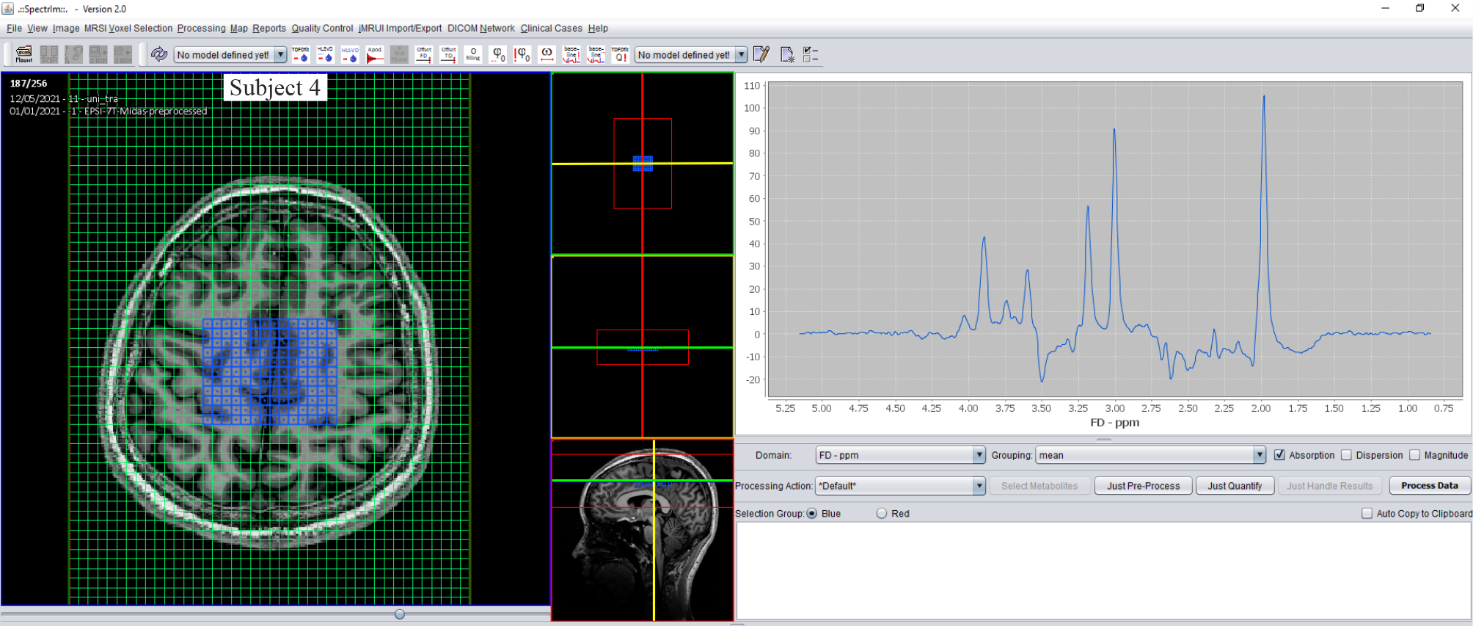
**

**Supporting Information Figure S6** **|** **The user interface (UI) of spectrIm-QMRS.** On the left is the T1-weighted MRI of a healthy volunteer (subject 4), and the displayed volume of the SLOW-full spectrum (blue grid, 14 × 11 × 1 matrix, 6.0 × 4.7 × 0.78 cm). On the right is the corresponding spectrum (Editing scheme 2, SLOW-full, TE = 68 ms).


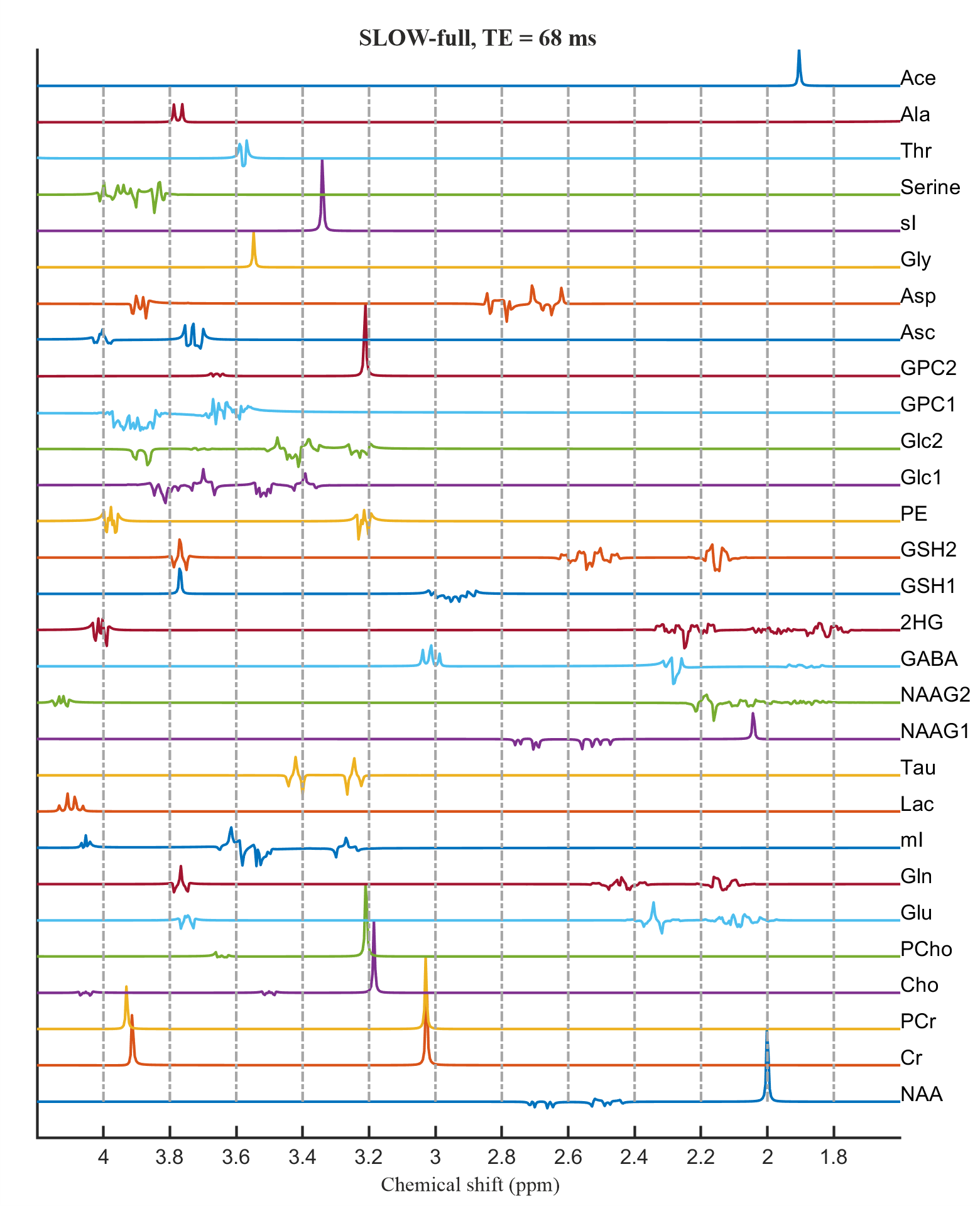


**Supporting Information Figure S**7 **|** **Simulation of the metabolite spectrum basis (Editing scheme 2, SLOW-full).** NAA (N‐acetyl aspartate), Cr (creatine), PCr (Phosphocreatine), Cho (choline), PCho (phosphocholine), Glu (glutamate), Gln (glutamine), mI (myo‐inositol), Lac (lactate), Tau (taurine), NAAG1 (N‐acetyl aspartyl glutamate-acetyl and aspartyl moiety), NAAG2 (N‐acetyl aspartyl glutamate-glutamate moiety), GABA (γ‐Aminobutyric acid), 2HG (2‐Hydroxyglutarate), GSH1 (glutathione-glycine and cysteine moiety), GSH2 (glutathione-glutamate moiety), PE (phosphoethanolamine), Glc1 (glucose, α‐anomer), Glc2 (glucose, β‐anomer), GPC1 (glycerophosphocholine-glycerol moiety), GPC2 (glycerophosphocholine-phosphocholinemoiety), Asc (ascorbic acid), Asp (aspartate), Gly (Glycine), sI (scyllo‐inositol), Serine, Thr (threonine), Ala (alanine), Ace (acetate). Note: it only shows the range from 1.6 – 4.2 ppm.


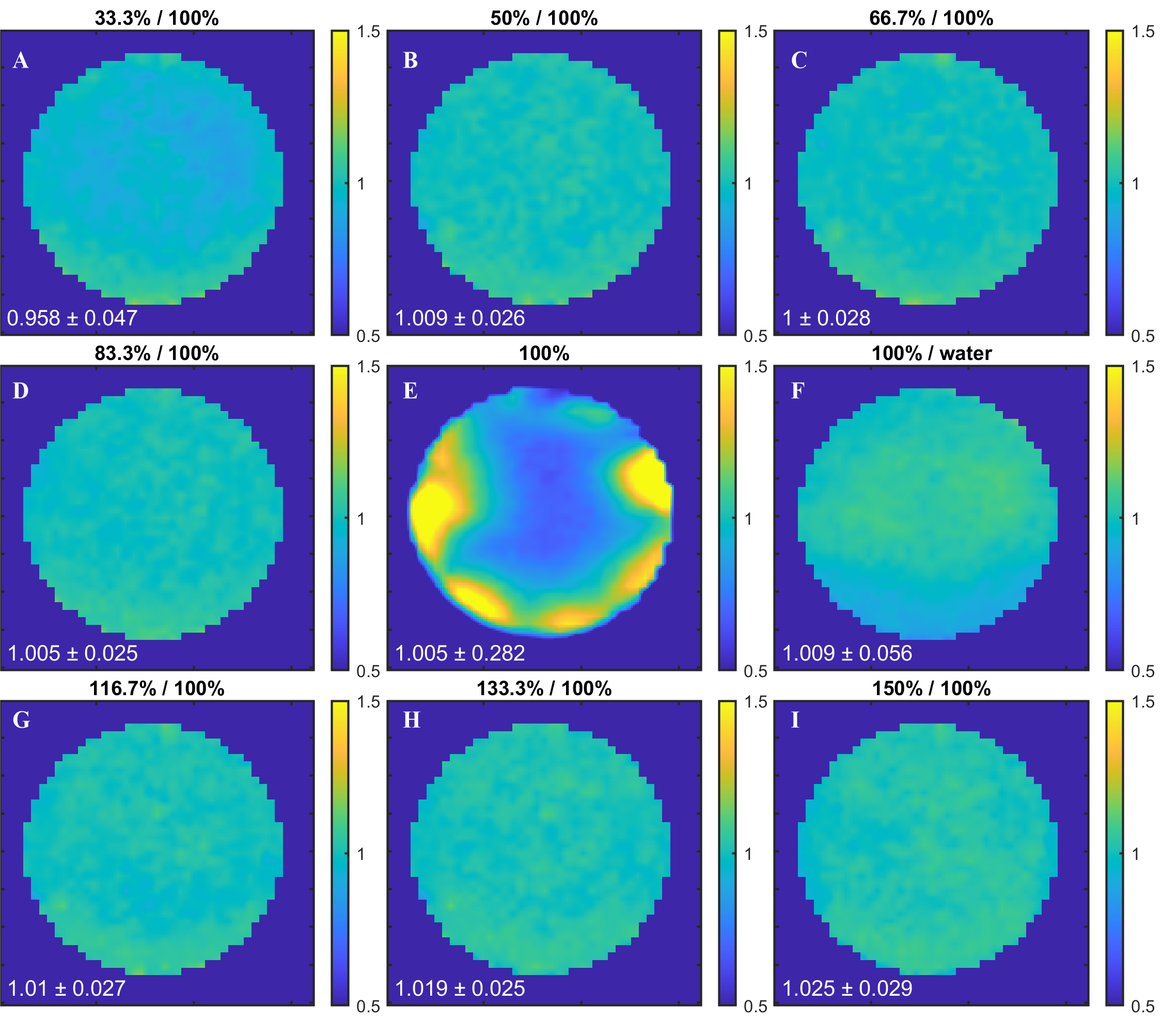


**Supporting Information Figure S8** **| In vitro Cr integration map using SLOW-partial (scheme 2) with different B1 amplitudes of 2π-CSAP. E)** Cr integration map with 100% B_1,max_ (550 Hz), 2D matrix was interpolated from 41 × 41 to 161 × 161. **F)** Cr map with B_1_ correction using water reference data. **A-D, G-I)** Cr maps with different B_1,max_ (from 33.3% to 150%). Note: the maps were divided by Cr map with 100% B_1,max_ instead of water reference. TE = 68 ms, TR = 1700 ms, FOV = 280 × 180 × 60 mm, matrix = 65 × 42 × 5 (4.3 × 4.3 × 12 mm resolution), and TA = 4:52 min. Post-processing: B_1_ correction using water refence data or Cr map with 100% B_1,max_. Mean and standard deviation indicated at the bottom left.


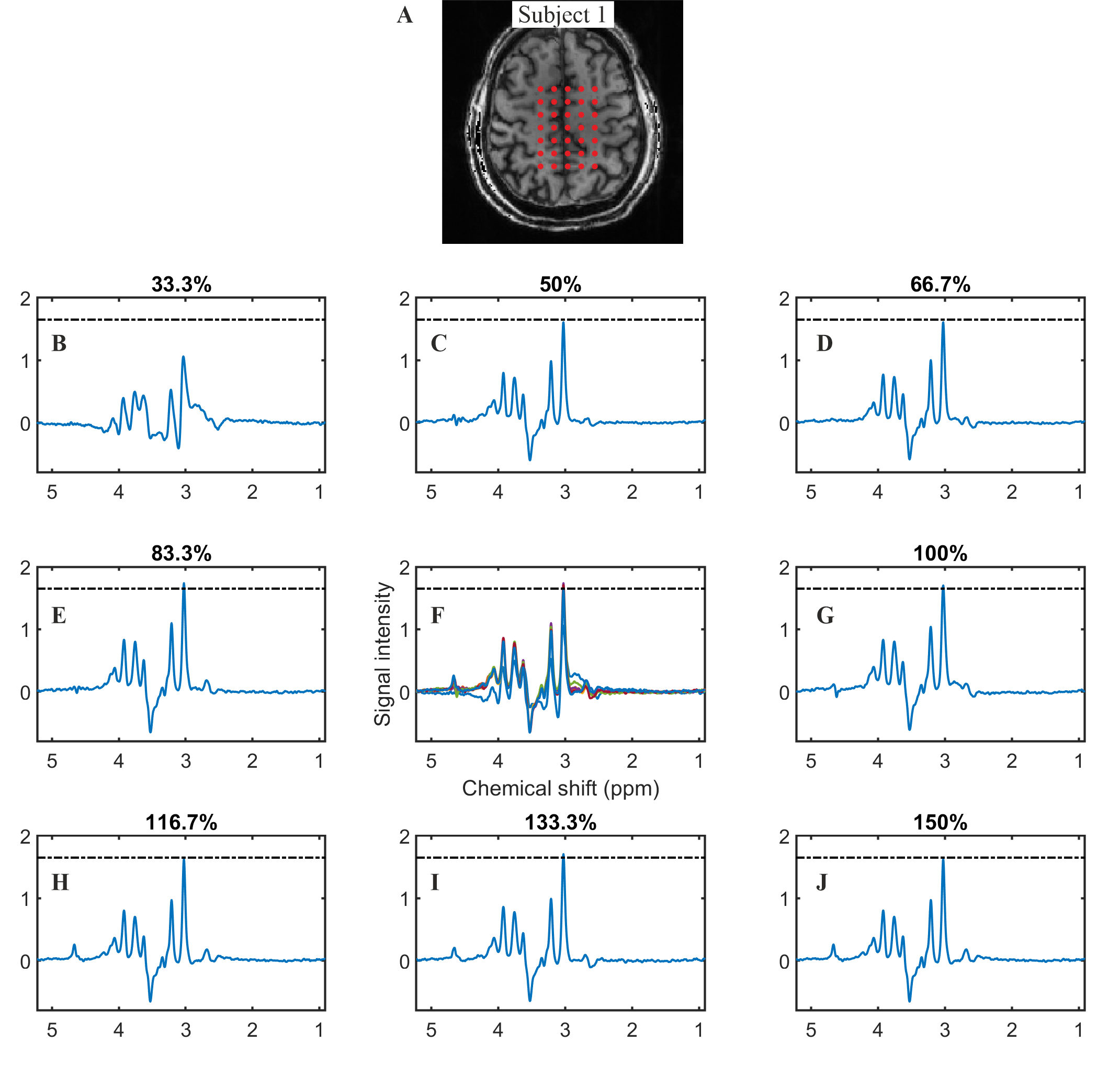


**Supporting Information Figure S9** **| In vivo SLOW-partial (scheme 2) with different B1 amplitudes of 2π-CSAP. A)** MRI and the selected voxels (red dots) for spectral. **B-E, G-J)** The average spectral of selected voxels in two slices (5 × 7 × 2 = 70 voxels, ~11 cm^3^) with different B1 amplitudes of 2π-CSAP, from 33% to 150%. The amplitude of 100% B_1,max_ is 550 Hz. **F)** Overlapping spectrum of B-E, G-J. TE = 68 ms, TR = 2200 ms, FOV = 280 × 180 × 60 mm, matrix = 65 × 23 × 5 (4.3 × 7.8 × 12 mm resolution), and TA = 3:33 min. Post-processing: B_1_ correction using water refence data.


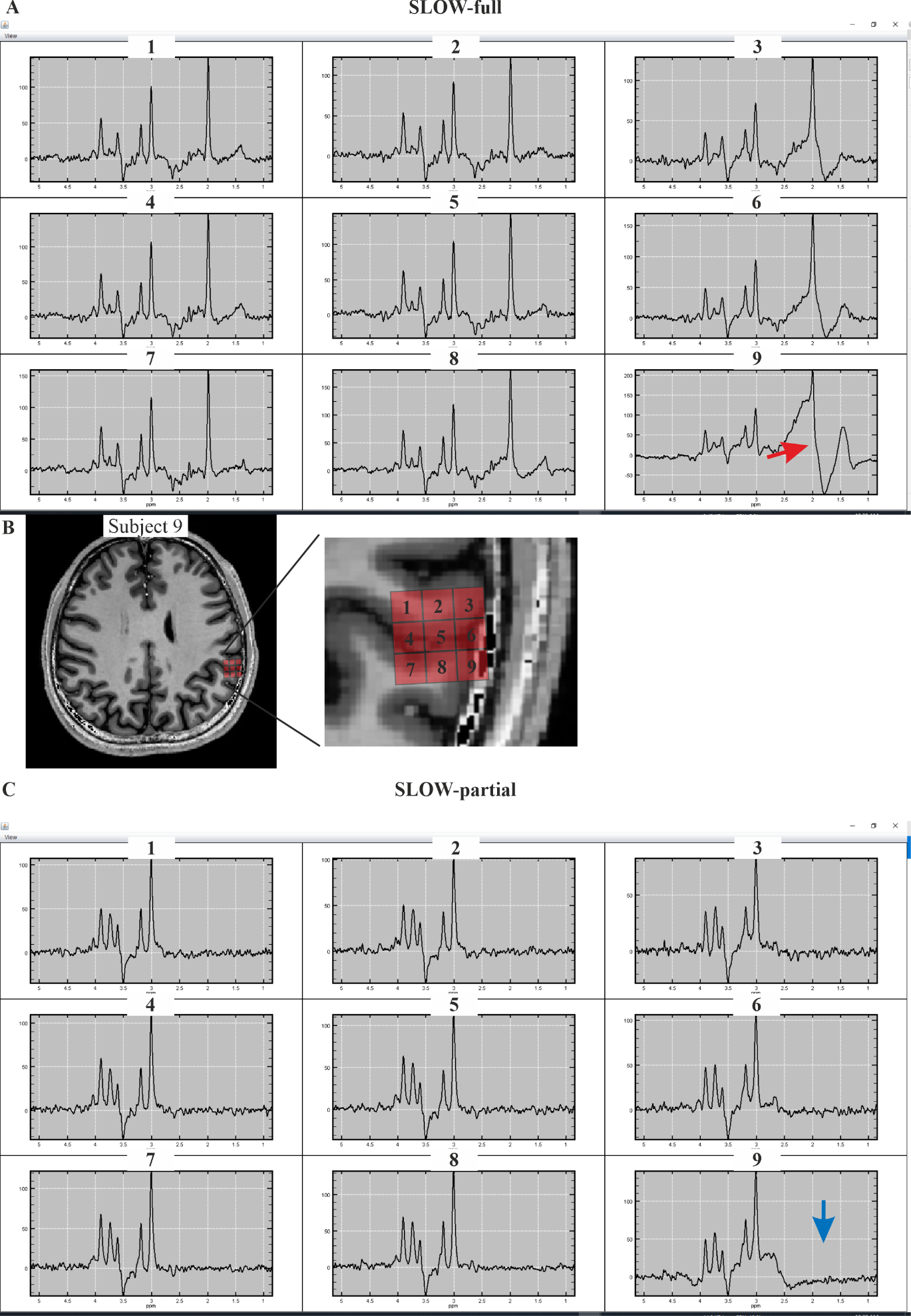


**Supporting Information Figure S10** **|** **SLOW-EPSI (scheme 2) of a healthy subject (#9) . A, C)** Matrix plot of 9 single voxel of SLOW-full and -partial (voxel size = 4.3 × 4.3 × 7 mm, interpolation matrix = 65 × 42 × 10), respectively. The corresponding location was indicated in B. **B)** MRI and the selected voxels (red grid) for spectral. TE = 68 ms, TR = 1500 ms, FOV = 280 × 180 × 70 mm, matrix = 65 × 23 × 9 (4.3 × 7.8 × 7.8 mm resolution), and TA = 4:32 min.


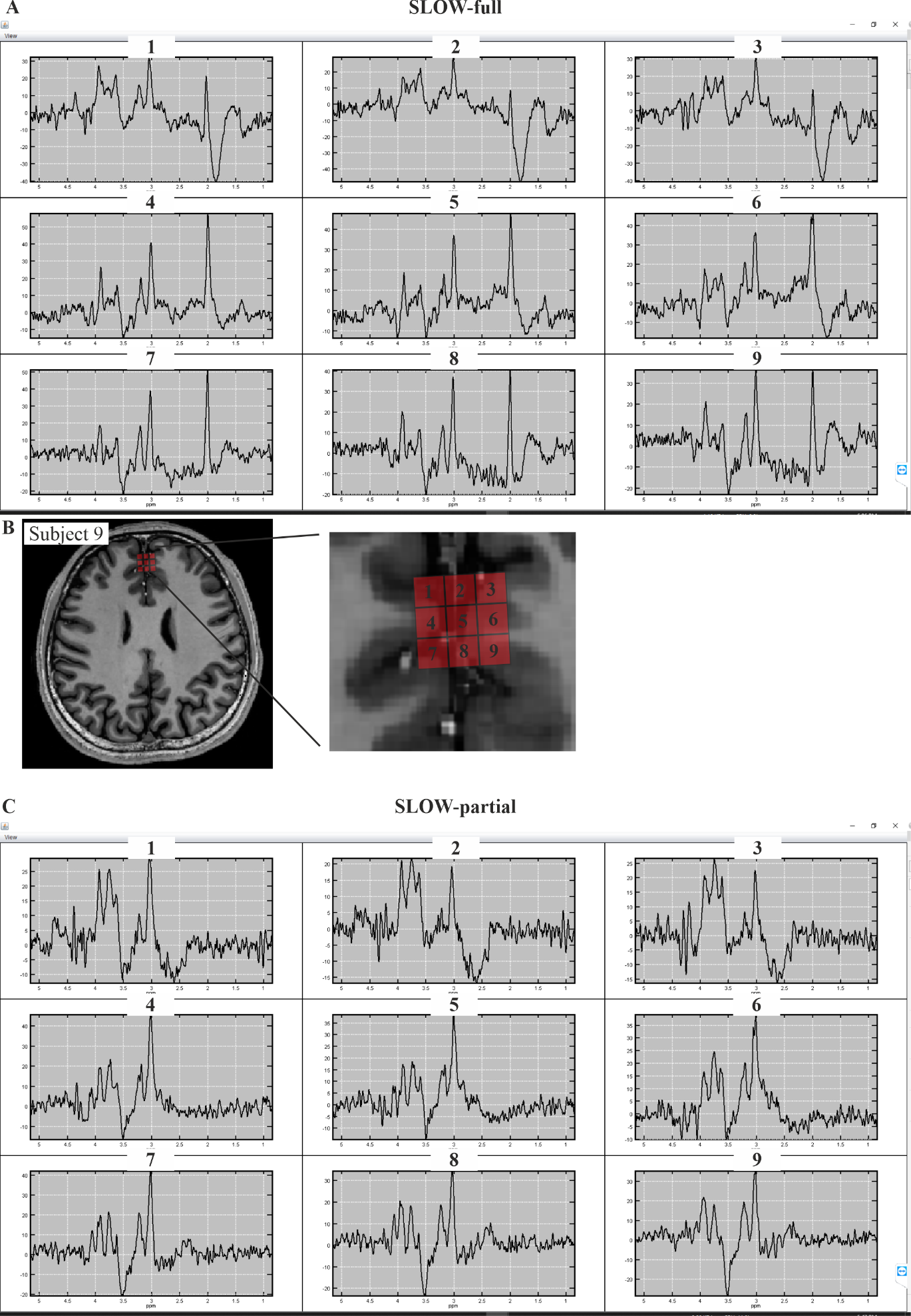


**Supporting Information Figure S11** **|** **SLOW-EPSI (scheme 2) of a healthy subject (#9). A, C)** Matrix plot of 9 single voxel of SLOW-full and -partial (voxel size = 4.3 × 4.3 × 7 mm, interpolation matrix = 65 × 42 × 10), respectively. The corresponding location was indicated in B. **B)** MRI and the selected voxels (red grid) for spectral. TE = 68 ms, TR = 1500 ms, FOV = 280 × 180 × 70 mm, matrix = 65 × 23 × 9 (4.3 × 7.8 × 7.8 mm resolution), and TA = 4:32 min.


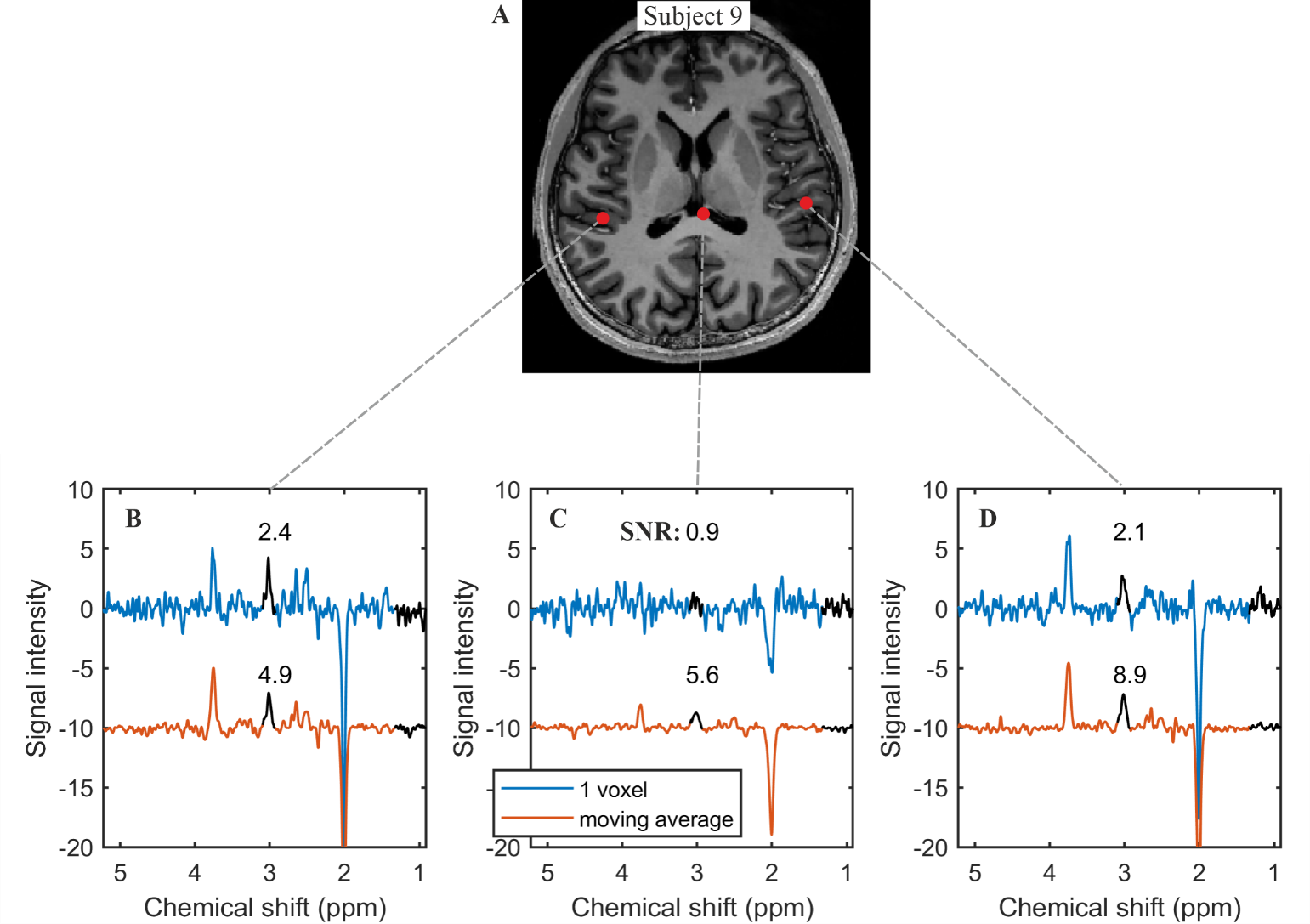


**Supporting Information Figure S12** **|** **GABA+ editing (scheme 2) of a healthy subject (#9). A)** MRI and the selected voxels (red dots) for spectral. **B-C)** The spectral of SLOW-difference with single voxel (blue, voxel size = 4.3 × 4.3 × 7 mm, interpolation matrix = 65 × 42 × 10) and moving average (orange, 43 voxels). The SNR of GABA+ was shown, and the spectral range used to calculate signal and noise level were marked as black lines at ~3.01 and ~1.1 ppm, respectively. TE = 68ms, TR = 1500 ms, FOV = 280 × 180 × 70 mm, matrix = 65 × 23 × 9 (4.3 × 7.8 × 7.8 mm resolution), and TA = 4:32 min. Post-processing: spline baseline correction, and moving average (43 voxels).


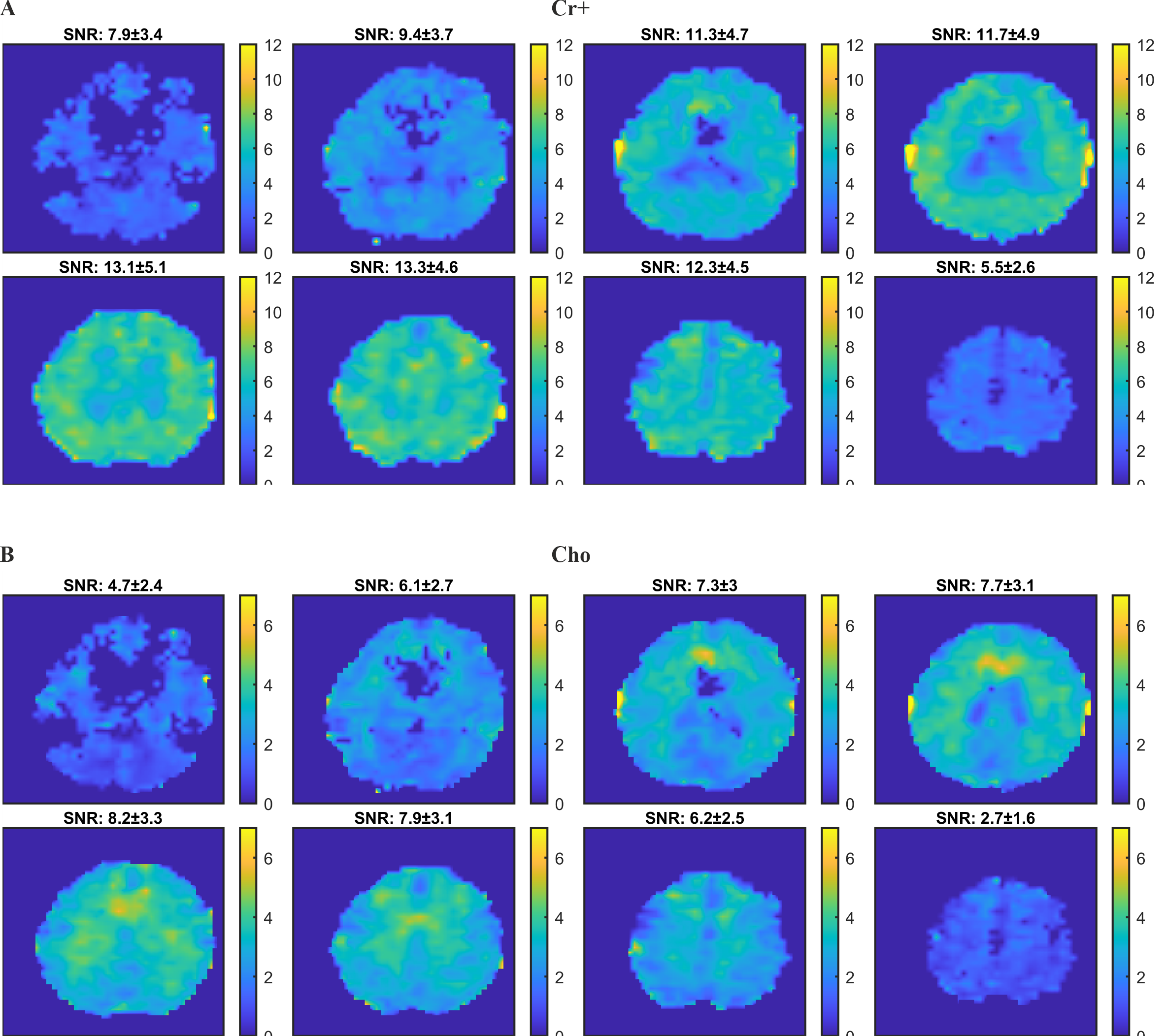


**Supporting Information Figure S13** **|** **Cr+ and Cho maps of a healthy subject (#9).** Gaussian fitting was performed at 3 and 3.2 ppm for Cr+ (Cr and GABA+) and Cho using the SLOW-partial dataset. 2D matrix was interpolated from 41 × 41 to 161 × 161. Post-processing: spline baseline correction and B_1_ correction using water refence data. Note: this is to show that SLOW-EPSI is able to produce whole brain metabolite maps, but it does not represent the final optimal quality using SLOW-EPSI. Further work on fitting and mapping is still in progress.


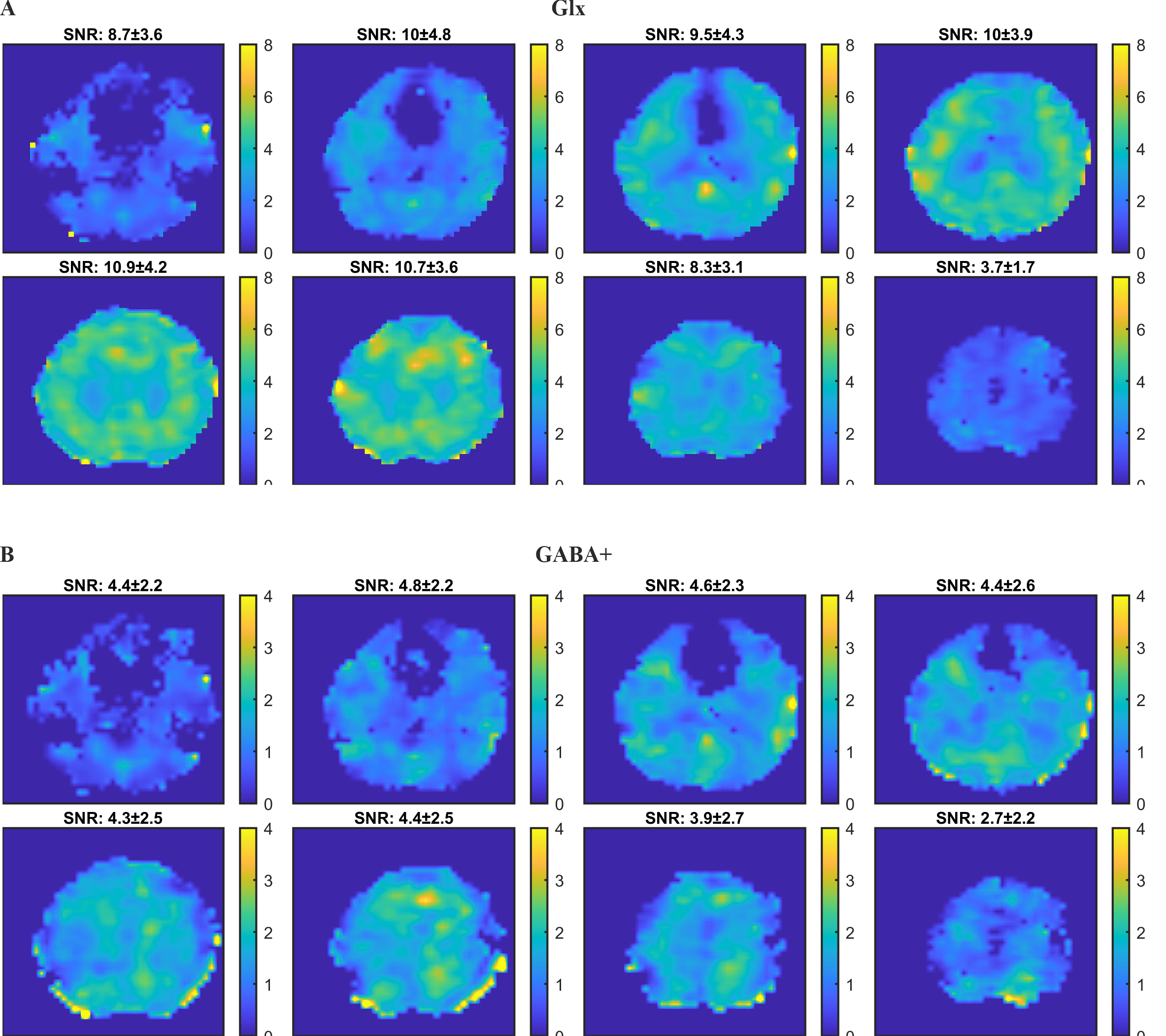


**Supporting Information Figure S14** **|** **Glx and GABA+ maps of a healthy subject (#9).** Gaussian fitting was performed at 3.75 and 3.01 ppm for Glx and GABA+ using the SLOW-difference dataset (SLOW-partial minus SLOW-full). 2D matrix was interpolated from 41 × 41 to 161 × 161. Post-processing: spline baseline correction, B_1_ correction using water refence data, and moving average (43 voxels). Note: this is to show that SLOW-EPSI is able to produce whole brain metabolite maps, but it does not represent the final optimal quality using SLOW-EPSI. Further work on fitting and mapping is still in progress.


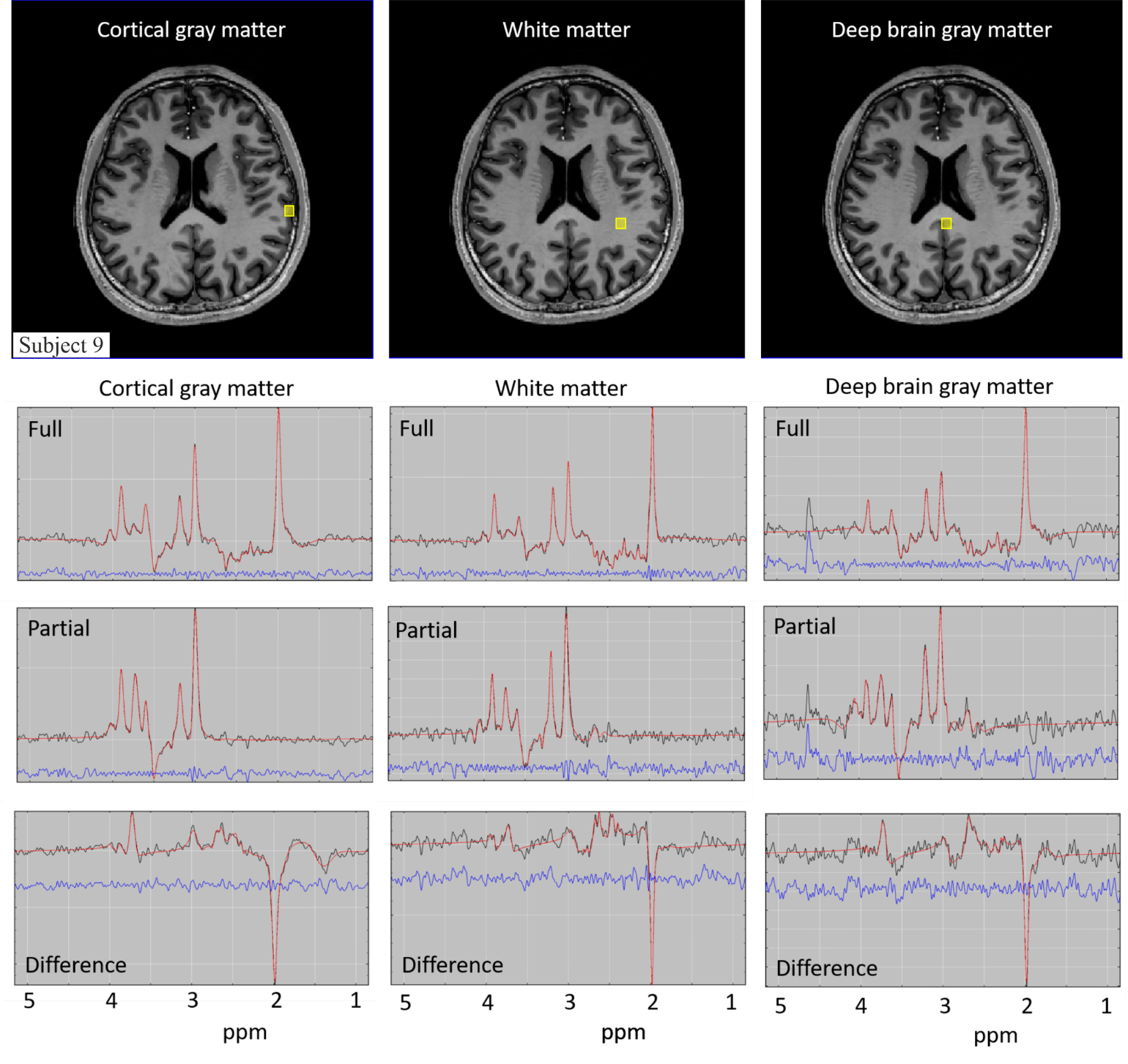


**Supporting Information Figure S15** **|** **Spectral fitting.** Three examples of the fit results obtained with TDFDFit assuming Voigt-lines for different locations. Post-processing: 5 voxels moving average.


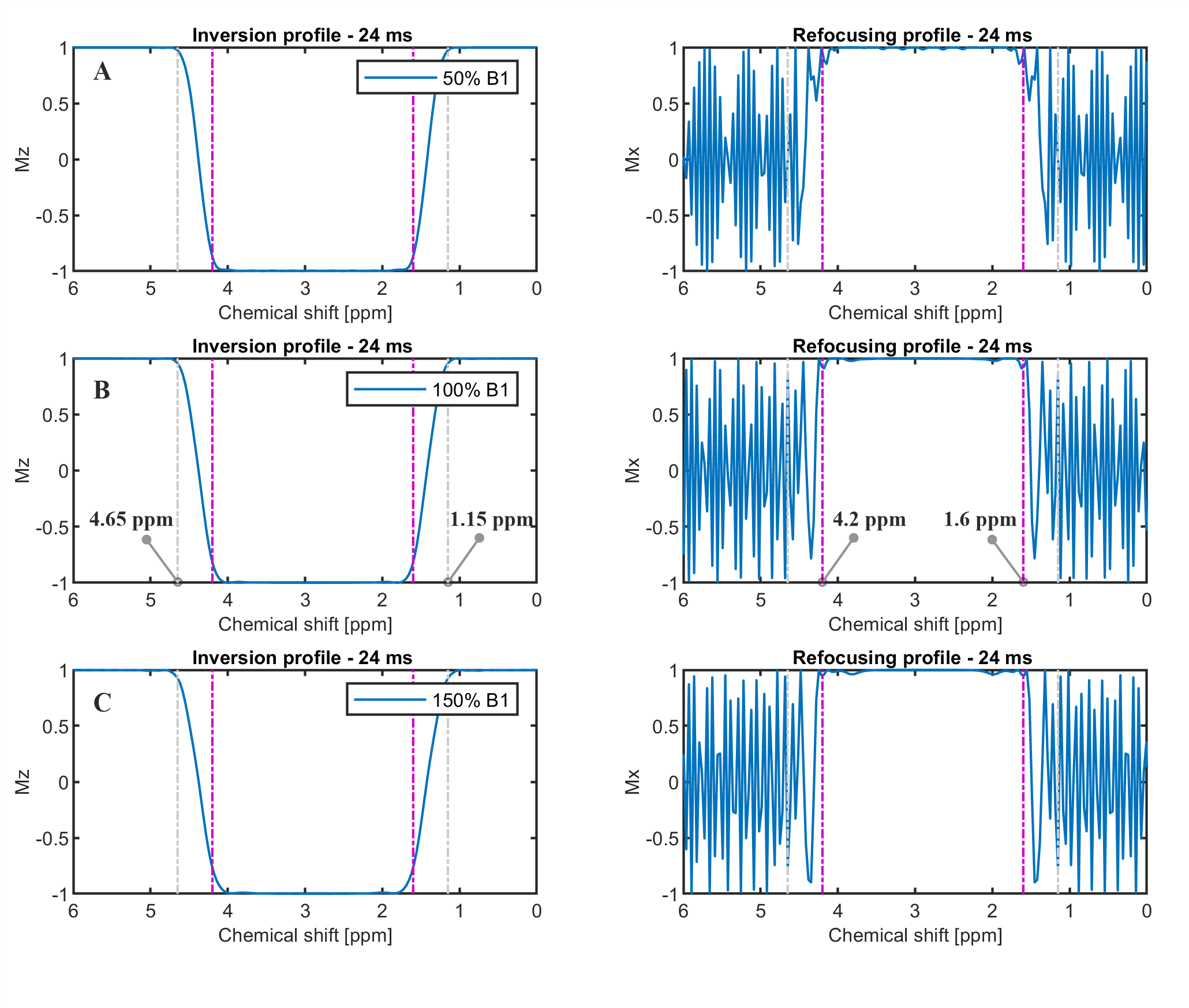


**Supporting Information Figure S16** **|** **Simulation for complex secant hyperbolic adiabatic pulse (scheme 2, editing-full). A-C)** The π-inversion and 2π-refocusing profiles with different B_1_ (50% - 150%).


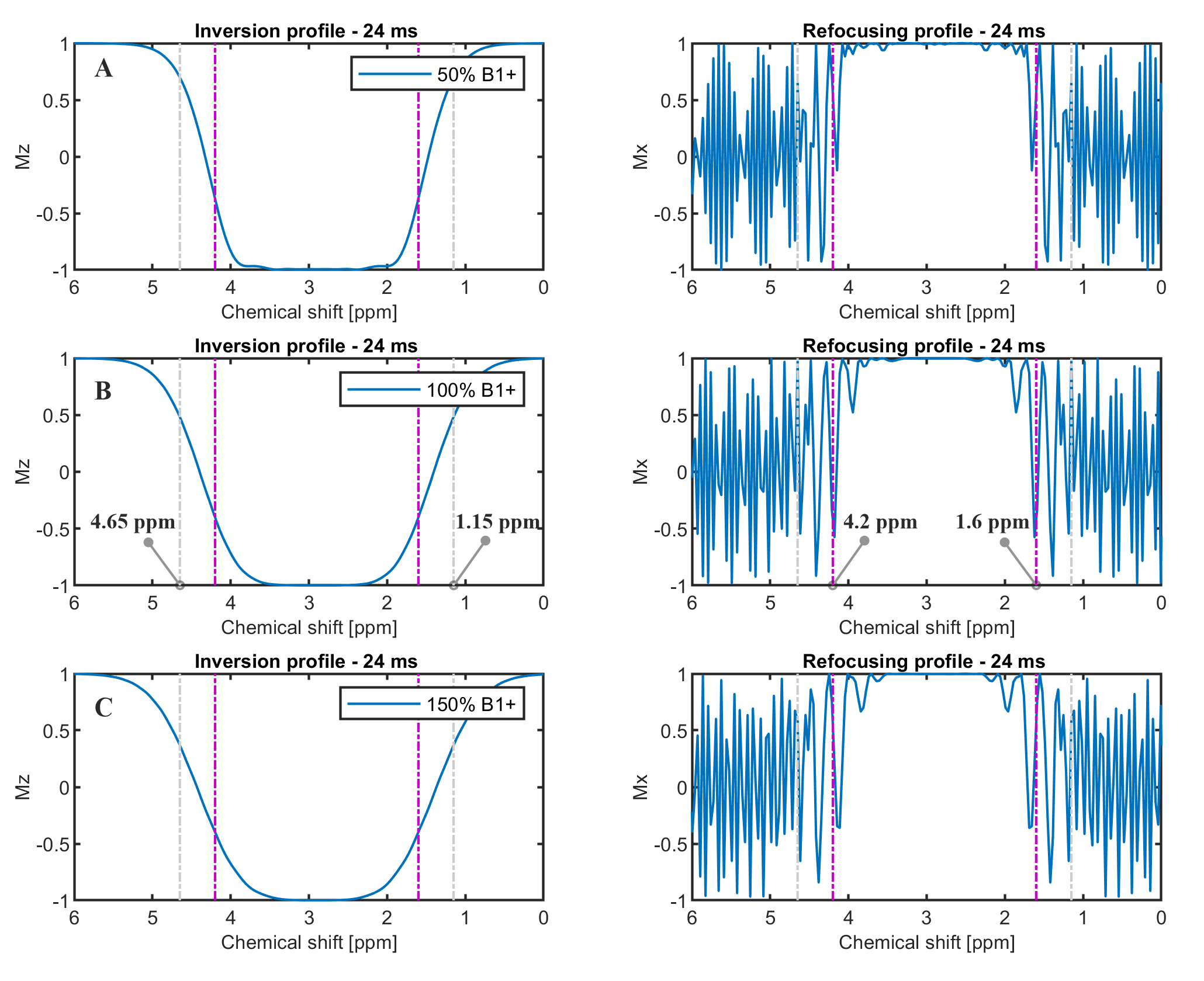


**Supporting Information Figure S17** **|** **Simulation for WURST-16 adiabatic pulse**^7,8^**. A-C)** The π-inversion and 2π-refocusing profiles with different B_1_ (50% - 150%). 24 ms duration, 880 Hz bandwidth, 100% B_1, max_ = 322 Hz, and k = 20 for phase modulation: $\phi(t)=k\left( \frac{2t}{\mathrm{dur}}-1 \right)^{2}, t is [0, 24] ms.$


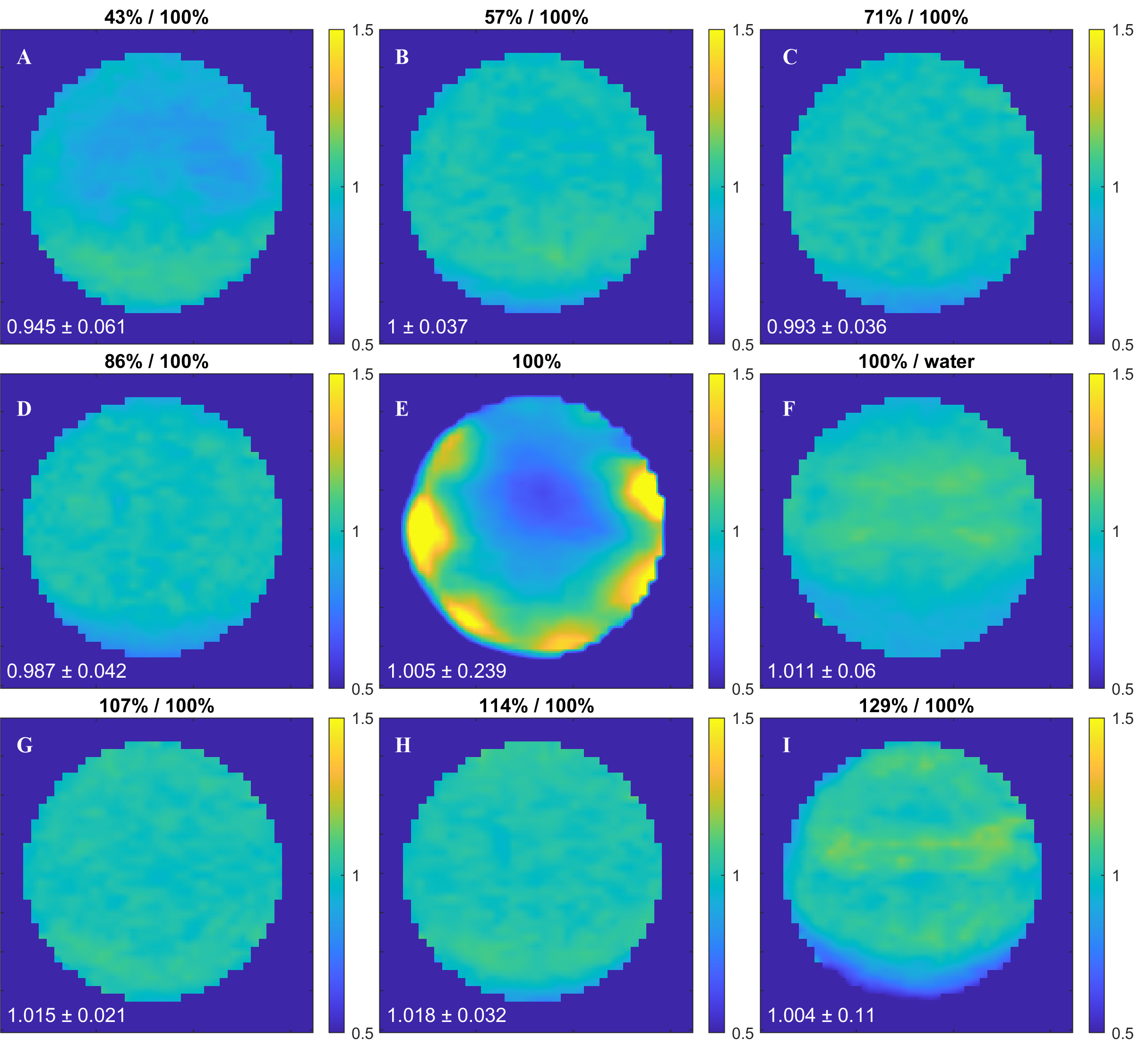


**Supporting Information Figure S18** **| In vitro integration map with different B1 amplitudes of 2π-CSAP (TE 30 ms). E)** Cr integration map with 100% B_1,max_ (786 Hz), 2D matrix was interpolated from 41 × 41 to 161 × 161. **F)** Cr map with B_1_ correction using water reference data. **A-D, G-I)** Cr maps with different B_1,max_ (from 43% to 129%). Note: the maps were divided by Cr map with 100% B_1,max_ instead of water reference. TE = 30 ms, TR = 1700 ms, FOV = 280 × 180 × 60 mm, matrix = 65 × 23 × 5 (4.3 × 7.8 × 12 mm resolution), and TA = 3:26 min. Post-processing: B_1_ correction using water refence data or Cr+ map with 100% B_1,max_. Mean and standard deviation indicated at the bottom left.

|  | $\Omega_{0} [Hz]$ | $\mu$ | $\beta$ | $f [Hz]$ | $t [ms]$ | Carrier frequency [ppm] |
| --- | --- | --- | --- | --- | --- | --- |
| Non-editing  (TE = 82ms) | 520 | 9.5 | $f\pi/\mu$ | 810 | [-15.5, 15.5] | 3.00 |
| Editing scheme 1-full | 520 | 9.5 | - | 810 | - | 3.00 |
| Editing scheme 1-partial | 520 | 9.5 | - | 810 | - | 2.00 |
| Editing scheme 2-full | 623 | 7.25 | - | 880 | [-12, 12] | 2.90 |
| Editing scheme 2-partial | 550 | 4.6 | - | 560 | - | 3.45 |
| Editing scheme 3-full | 562 | 7.45 | - | 800 | [-17.5, 17.5] | 3.00 |
| Editing scheme 3-full | 412 | 6.78 | - | 560 | - | 2.60 |
| Inversed recovered part 1 | 93 | 9.15 | - | 160 | [-80, 0] | 1.57 |
| Inversed recovered part 2 | 93 | 1.4 | - | 80 | [0, 20] |  |

**Supporting Information Table S1** **| The RF-pulse parameters of adiabatic pulses used.**

| Metabolite | Editing difference  (cortex/white matter/deep brain) | Partial  (cortex/white matter/deep brain) | Full  (cortex/white matter/deep brain) |
| --- | --- | --- | --- |
| NAA | 1.03/1.58/1.51 | - | 1.63/1.21/1.03 |
| Glx | 2.34/5.99/4.68 | 1.49/1.67/3.21 | 7.16/10.9/16.7 |
| GABA | 5.42/5.98/4.46 | 17.5/10.8/14.39 | - |
| Cho | - | 1.32/1.15/2.01 | 1.78/1.92/1.56 |
| Cr | - | 4.47/2.39/5.02 | 1.13/1.19/0.95 |
| mI | - | 4.27/4.25/4.57 | 8.97/5.57/3.15 |

**Supporting Information Table S2** **| CR-MVB Relative %-errors.** Typical CR-MVB errors found for cortical GM, WM, and deep brain GM. “-” means the corresponding metabolite fitting did not apply.
